# Contribution of Endothelial Dysfunction to Cancer Susceptibility and Progression: A Comprehensive Narrative Review on the Genetic Risk Component

**DOI:** 10.3390/cimb46050292

**Published:** 2024-05-16

**Authors:** Inês Guerra de Melo, Valéria Tavares, Deolinda Pereira, Rui Medeiros

**Affiliations:** 1 Molecular Oncology and Viral Pathology Group, Research Center of IPO Porto (CI-IPOP)/Pathology and Laboratory Medicine Dep., Clinical Pathology SV/RISE@CI-IPOP (Health Research Network), Portuguese Oncology Institute of Porto (IPO Porto)/Porto Comprehensive Cancer Centre (Porto. CCC), 4200-072 Porto, Portugal; ines.melo@ipoporto.min-saude.pt (I.G.d.M.); valeria.tavares@ipoporto.min-saude.pt (V.T.); 2 Faculty of Medicine of University of Porto (FMUP), 4200-072 Porto, Portugal; 3 ICBAS—Instituto de Ciências Biomédicas Abel Salazar, Universidade do Porto, 4050-313 Porto, Portugal; 4 Oncology Department, Portuguese Oncology Institute of Porto (IPO Porto), 4200-072 Porto, Portugal; dpereira@ipoporto.min-saude.pt; 5 Faculty of Health Sciences, Fernando Pessoa University, 4200-150 Porto, Portugal; 6 Research Department, Portuguese League Against Cancer (NRNorte), 4200-172 Porto, Portugal

**Keywords:** thrombosis, venous thromboembolism, neoplasm, genetic polymorphism, endothelium

## Abstract

Venous thromboembolism (VTE) is a challenging clinical obstacle in oncological settings, marked by elevated incidence rates and resulting morbidity and mortality. In the context of cancer-associated thrombosis (CAT), endothelial dysfunction (ED) plays a crucial role in promoting a pro-thrombotic environment as endothelial cells lose their ability to regulate blood flow and coagulation. Moreover, emerging research suggests that this disorder may not only contribute to CAT but also impact tumorigenesis itself. Indeed, a dysfunctional endothelium may promote resistance to therapy and favour tumour progression and dissemination. While extensive research has elucidated the multifaceted mechanisms of ED pathogenesis, the genetic component remains a focal point of investigation. This comprehensive narrative review thus delves into the genetic landscape of ED and its potential ramifications on cancer progression. A thorough examination of genetic variants, specifically polymorphisms, within key genes involved in ED pathogenesis, namely *eNOS*, *EDN1*, *ACE*, *AGT*, *F2*, *SELP*, *SELE*, *VWF*, *ICAM1*, and *VCAM1*, was conducted. Overall, these polymorphisms seem to play a context-dependent role, exerting both oncogenic and tumour suppressor effects depending on the tumour and other environmental factors. In-depth studies are needed to uncover the mechanisms connecting these DNA variations to the pathogenesis of malignant diseases.

## 1. Introduction

Venous thromboembolism (VTE), also referred to as venous thrombosis, is a prevalent and intricate cardiovascular condition. The disease has two main manifestations: when the thrombus first forms in a deep vein—deep vein thrombosis (DVT)—followed by its migration into the bloodstream and subsequent lodging in the lungs—pulmonary embolism (PE) [1]. In Europe, VTE affects around one to two individuals per 1000 annually [2]. Although the incidence rate in the United States of America (USA) is greatly similar, it varies significantly from a global perspective, indicating a potential regional influence on the occurrence of thrombotic events. Indeed, VTE has been associated with multiple risk determinants, comprising acute (e.g., surgery) and subacute (e.g., oral contraceptive use) triggers; basal/genetic (e.g., genetic polymorphisms, such as Factor V Leiden (*F5* rs6025) and Prothrombin G20210A (*F2* rs1799963)) and acquired (e.g., autoimmune diseases) risk factors [2,3]. One acquired risk factor of VTE that warrants prominent consideration is cancer. With an estimated annual incidence of VTE at 0.5% among cancer patients, compared to 0.1% in the general population, these statistics underscore the markedly heightened vulnerability to venous thrombogenesis among individuals with malignant diseases [4]. In recent years, the link between cancer physiopathology and VTE has attained increasing attention, leading to the emergence of the concept of cancer-associated thrombosis (CAT). This constitutes a bidirectional relationship, wherein both cancer and VTE serve as mutual risk factors for each other, as well as exert a significant impact on each other’s mortality rates [5]. Compared to VTE in the general population, CAT seems to be a distinct and more complex disorder. Mechanistically, tumour cells produce pro-coagulant, anti-fibrinolytic and pro-inflammatory substances, which trigger pro-thrombotic and pro-inflammatory cascades leading to venous thrombogenesis [4].

**Figure 1:**
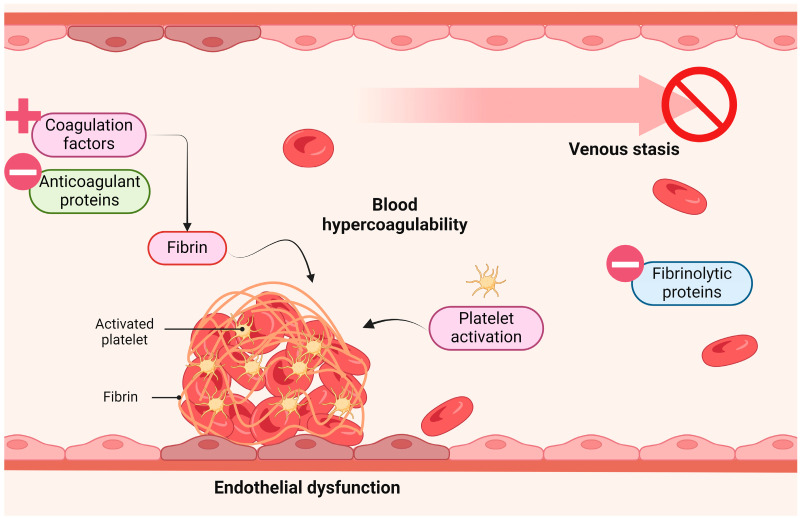
Virchow triad. Venous thrombosis is suggested to be promoted by three important factors: blood hypercoagulability, venous stasis and endothelial dysfunction. Figure created with Biorender.com (accessed on 13 April 2024).

The pathogenesis of VTE, both in the general population and among cancer subjects, can be explained by the Virchow Triad, which integrates three promoting factors: stasis of blood flow, blood hypercoagulability, and endothelial dysfunction (ED) (Figure 1) [4,6]. ED refers to an alteration in the normal function of the endothelial cells (ECs) lining the interior of blood vessels. The first step of this disorder is endothelial stimulation (type I activation) followed by delayed endothelial activation (type II activation), both reversible upon cessation of the stimulus. In advanced stages, ED also encompasses EC apoptosis and necrosis, which leads to endothelial detachment, giving rise to circulating endothelial cells (CECs) [7]. Apart from contributing to VTE, ED is a critical factor in the pathogenesis of other cardiovascular and metabolic diseases, including atherosclerosis, hypertension, coronary artery disease, chronic heart failure, peripheral artery disease, diabetes, and chronic renal failure [8,9,10]. Importantly, a relevant bridge to cancer is also formed as the pro-inflammatory state of ED promotes tumour growth and progression. Additionally, the inhibition of vasodilation (characteristic of ED) supports cell proliferation and anti-apoptotic responses, reinforcing its association with cancer [11,12].

Like VTE, ED presentation can also be influenced by genetic polymorphisms, which are DNA variations present in greater than 1% of a given population. These variations include single-nucleotide polymorphisms (SNPs), copy number variations (CNVs), insertions and deletions (Indels), and tandem repeats [13,14]. Starting with SNPs, they represent genetic alterations characterised by single nucleotide substitutions [15]. CNVs arise from the deletion or duplication of DNA segments, ranging from kilobases to megabases, leading to a varied number of copies of a specific DNA sequence on homologous chromosomes [16]. In contrast, indels are small insertions or deletions of nucleotides in the DNA sequence [17]. Regarding tandem repeats, these genetic variations comprise repetitive DNA sequences spanning one or more nucleotides within both coding and non-coding regions. Their classification depends on the length of the repeated sequence. Namely, simple sequence repeats (SSRs), also known as short tandem repeats (STRs), consist of short repeating units (two to six nucleotides). SSRs represent a subset of a variable number of tandem repeats (VNTRs), characterised by their varying lengths [18]. Overall, genetic polymorphisms have the potential to modulate gene expression, disrupt gene function and alter protein-coding sequences, thereby affecting protein levels and/or activity. Consequently, these DNA variations can modulate the susceptibility to several disorders, including ED and its manifestations (e.g., VTE) [14].

Considering the roles of ED in cancer-related thrombogenesis and tumorigenesis, it is important to explore how genetic determinants implicated in this disorder could aid in the identification of at-risk populations and pinpoint potential therapeutic targets for a more personalised treatment in Oncology [14]. Given the implications for clinical application, this thorough narrative review seeks to delve into the influence of genetic variations linked to ED on tumorigenesis and cancer patient’s prognosis. The review concentrates on examining polymorphisms in pivotal ED-related genes such as *endothelial nitric oxide synthase* (*eNOS*), *endothelin 1* (*EDN1*), *angiotensin I converting enzyme* (*ACE*), *angiotensinogen* (*AGT*), *coagulation factor 2* (*F2*), *selectin P* (*SELP*), *selectin E* (*SELE*), *von Willebrand factor* (*VWF*), *intercellular adhesion molecule 1* (*ICAM1*), and *vascular cell adhesion molecule 1* (*VCAM1*). A thoughtful research was conducted by reviewing the PubMed database’s occurrences until 6th March 2024 using different combinations of keywords: “SNP”, “SNPs”, “polymorphism”, “polymorphisms”, “cancer”, “*eNOS*”, Endothelin-1”, “ET-1”, “Angiotensin II”, “AGT”, “ACE” and “Angiotensin Converting Enzyme”, “F2”, “Prothrombin”, “SELP”, “P-selectin”, “SELE”, “E-selectin”, “E-selectin”, “Von Willebrand factor”, “VWF”, “CD54”, “ICAM1”, “ICAM-1”, “VCAM1”, “VCAM-1” and “CD106”. Only studies with significant associations were selected. Additionally, matching publications were cross-referenced and screened for pertinent bibliographic references. Studies were excluded if the polymorphisms lacked functional relevance and/or the associations were observed solely considering specific therapeutic interventions. A total of 826 articles underwent review, resulting in the selection of 149 papers that met the inclusion and exclusion criteria.

## 2. Vascular Homeostasis

**Figure 2:**
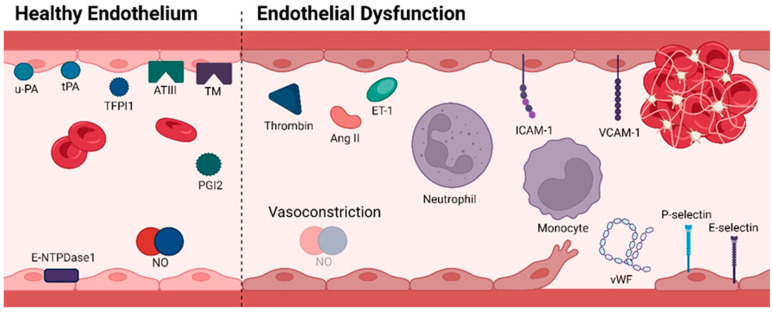
Molecular profile of a healthy endothelium characterized by anti-thrombotic mechanisms (**left**); Molecular profile of endothelial dysfunction with the display of adhesive, pro-inflammatory, and pro-clotting properties (**right**). Abbreviations: ATIII, antithrombin III; ET-1, Endothelin-1; E-NTPDase1, ectonucleoside triphosphate diphosphohydrolase-1; ICAM-1, intercellular adhesion molecule-1; NO, nitric oxide; PGI2, prostaglandin I2; TFPI1, tissue factor pathway inhibitor 1; TM, thrombomodulin; tPA, tissue-type plasminogen activator; u-PA, urokinase-type plasminogen activator; VCAM-1, vascular cell adhesion molecule-1; vWF, von Willebrand factor. Figure created with Biorender.com (accessed on 13 April 2024).

Gatekeeping the integrity of ECs is crucial for human well-being and illness management as these cells are responsible for vascular tone regulation, haemostasis and thrombosis control, cellular adhesion, smooth muscle cell proliferation, and vascular inflammation [6,19]. Under physiological conditions, ECs mediate multiple anti-coagulant and anti-platelet aggregation processes, restricting coagulation to only vascular sites where needed, thus preventing disseminated thrombotic complications (Figure 2) [20]. They do so by continuously expressing and/or releasing components that block platelet activity (prostacyclin (prostaglandin I2 (PGI2), nitric oxide (NO) and ectonucleoside triphosphate diphosphohydrolase-1 (E-NTPDase1)), inhibit coagulation progression (antithrombin III (ATIII)), thrombomodulin (TM) and tissue factor pathway inhibitor 1 (TFPI1)) and promote fibrinolysis (urokinase-type plasminogen activator (u-PA) and tissue-type plasminogen activator (tPA)) [21,22,23,24]. In opposition, when the vascular endothelium is disrupted, ECs shift to an adhesive, pro-inflammatory and pro-clotting phenotype [21]. The initial response to vascular damage is vasoconstriction, which slows the blood flow to prevent excessive blood loss. This mechanism is the basis for blood coagulation [25]. Parallelly, induced by pro-inflammatory cytokines, ECs express cell-surface adhesion molecules essential for the recruitment and attachment of immune cells against possible pathogens [26]. Once an immune barrier is established and haemostasis is restored, a process of vascular repair is initiated [27]. However, under pathological conditions (such as hyperhomocysteinaemia, hyperglycaemia, hypercholesterolaemia and accumulation of NO inhibitors), the endothelium loses its natural properties, shifting towards reduced vasodilation, inflammation and thrombosis, which overall defines ED [28]. Essentially, a dysfunctional endothelium arises when there is an imbalance between endothelium-derived relaxation (EDRFs) and constriction (EDCFs) factors. The former includes NO, prostacyclin and endothelium-derived hyperpolarizing factor (EDHF), while endothelin (ET-1), angiotensin II (Ang II), thrombin and thromboxane A2 (TXA2) represent EDCFs [6]. It is worth noting that prostacyclin, EDHF and TXA2 fall outside the scope of this review.

### Nitric Oxide (NO)

The most well-defined EDRF is NO, which is the protector of the vascular wall, with anti-inflammatory and antioxidant properties [6,10,29,30]. In addition to being a potent vasodilator gatekeeping endothelial health, NO is also a concentration-dependent cell proliferation and apoptosis modulator, as low relative concentrations appear to promote cell proliferation and anti-apoptotic responses and vice-versa [12,31,32]. Furthermore, as previously mentioned, it possesses platelet inhibitor properties [21]. Consequently, a decrease in NO bioavailability usually occurs in tandem with a pro-thrombotic and pro-inflammatory cascade and a less flexible endothelial state [6,28,30].

Deficiencies of NO can be caused by alterations in nitric oxide synthase 3 (NOS3), also known as eNOS [33]. The linkage between NO, ED and cancer is reinforced by the cell proliferation and anti-apoptotic pathways activated when this vasodilator is reduced, which enables tumour spread, angiogenesis and metastasis [34]. According to the literature, there is a total of 168 genetic polymorphisms located within or close to *NOS3*, of which three have emerged as particularly noteworthy due to their shared impact on reducing NO levels and their established associations with cancer: rs2070744, rs1799983, and rs869109213 (Table 1) [33].

Regarding rs2070744 (T>C), this intronic variant consists of the substitution of thymine (T) to cytosine (C) at codon -786 in the 5′-flanking region of *NOS3*. This alternation leads to diminishing gene promoter activity, with consequent serum NO reduction, enabling proliferation pathways and inhibiting tumour cell apoptosis [12,35,36]. To date, many meta-analyses associated rs2070744 with the risk of overall cancer, particularly among individuals of Caucasian descent. Further clustering by cancer type links the C allele (the minor and also ancestral allele) to a higher risk of breast (BC), prostate (PCa), and bladder (BLCA) cancers [33,36,37,38,39,40,41]. In a study regarding oral squamous cell carcinoma (OSCC), individuals with the TC genotype faced an increased likelihood of progressing to an advanced clinical stage (III/IV) compared to those with the TT genotype [42]. Similarly, BC patients carrying the C allele exhibited a significantly higher risk of disease recurrence or mortality compared to those with the TT genotype [12]. Carriers of the C allele are also more prone to colorectal cancer (CRC) [43]. Likewise, the CC genotype was associated with a five-fold increased risk for gastric cancer (GC) development [34]. On the other hand, regarding PCa in the Turkish population, the C allele was found to be less prevalent among patients compared to healthy controls, suggesting a protective effect of this allele [44,45]. Moreover, the C allele among uterine cervical cancer (UCC) patients was associated with a reduced risk of advancing to later disease stages, invasion of the parametrium, and metastasis to pelvic lymph nodes [46].

Another important polymorphism of *NOS3* is rs1799983 (G>T). This missense SNP leads to a glutamate-to-aspartate (Glu-to-Asp) substitution at position 298 in exon 7 [36,47]. This variant is linked to a substantial reduction in eNOS enzyme activity. Notably, this SNP exhibited associations with PCa, BLCA, and BC [33,36,38,39,40,45]. Concerning BC development, the effect of the T allele (minor allele) depends on the menopause status, exerting a protective effect on postmenopausal women [48]. Contrariwise, the presence of this allele was associated with an increased susceptibility to CRC [43]. Different populational studies have suggested a negative effect of the T allele concerning CRC, BLCA and endometrial carcinoma (EMCA) [47,49,50]. Furthermore, two investigations have identified noteworthy associations between this SNP and lung cancer (LC) and urothelial cell carcinoma (UC), respectively. The first one demonstrated a link to EGFR-mutated lung adenocarcinomas, particularly with exon 19 in-frame deletions, suggesting this SNP as a potential predictor of tumour invasiveness and responsiveness to therapy [51]. The second study denoted a propensity for increased tumour size development among UC patients carrying the rs1799983 T allele [52].

The variant rs869109213 (4a/b) is a VNTR polymorphism (27 bp) in the intron 4 of *NOS3* consisting of two alleles: 4a (with four repeats) and 4b (with five repeats). This DNA variation is linked to modified eNOS activity, affecting the baseline production of plasma NO. Specifically, the 4a allele carries present lower NO levels compared to those with the 4b/4b genotype [53]. Similarly to rs2070744, rs869109213 is associated with overall cancer risk in Caucasians, particularly PCa [33,37,38,40]. The 4a allele is linked to a higher risk of CRC in an early-onset (under 60 years old) [54]. Moreover, the heterozygous genotype (4a/4b) was found to be more common in BC patients when compared with a control group [55]. In the context of LC, a noteworthy association was also identified, however, linking the 4b allele to a higher risk for disease development [56]. In another study, the rs869109213 4a/4b genotype in combination with the rs2070744 CC genotype, as well as the C/G/4b haplotype for rs2070744/rs1799983/rs869109213 exhibited a 21-fold and 11-fold escalation in the risk of developing OSCC, respectively [57].

**Table 1:** Epidemiological studies on the role of *NOS3* polymorphisms on cancer susceptibility and progression.

First Author (Year)	Country/Ethnic Background	Population Characteristics	Study Design	Studied Polymorphisms
Choi et al. (2006) [12]	South Korea/Unclear	1039 BC patients 995 non-cancer controls	Cohort study	rs2070744 rs1799983
Lu et al. (2006) [41]	USA/non-Hispanic Caucasian	421 BC patients 423 non-cancer controls	Case–control study	rs2070744 rs1799983 rs869109213
Yeh et al. (2009) [54]	Taiwan/Taiwanese	727 CRC patients 736 healthy controls	Case–control study	rs2070744 rs1799983 rs869109213
Oztürk et al. (2011) [47]	Turkey/Turkish	89 EMCA patients 60 total hysterectomy controls	Case–control study	rs1799983 rs869109213
Arıkan et al. (2012) [49]	Turkey/Turkish	84 CRC patients 99 healthy controls	Case–control study	rs1799983
Jang et al. (2013) [43]	South Korea/Korean	528 CRC patients 509 healthy controls	Case–control study	rs2070744 rs1799983 rs869109213
Ramírez-Patiño et al. (2013) [55]	Mexico/Mexican	429 BC patients 281 healthy women	Case–control study	rs869109213
Wu et al. (2014) [36]	Mixed	4169 cancer cases and 4185 controls (rs2070744) 7775 cancer cases and 7817 controls (rs1799983) 3430 cancer cases and 3842 controls (rs869109213)	Meta-analysis	rs2070744 rs1799983 rs869109213
Zhang et al. (2014) [37]	Mixed	4220 cancer cases and 4016 controls (rs2070744) 8359 cancer cases and 9575 controls (rs1799983) 2873 cancer cases and 3338 controls (rs869109213)	Meta-analysis	rs2070744 rs1799983 rs869109213
Gao et al. (2015) [33]	Mixed (meta-analysis) Han Chinese (case–control)	873 BC patients 1034 healthy women (case–control)	Meta-analysis Case–control study	rs2070744 rs1799983 rs869109213
Krishnaveni et al. (2015) [34]	India/South Indian	150 GC patients 150 healthy controls	Case–control study	rs2070744
Polat et al. (2015) [50]	Turkey/Turkish	75 BLCA patients 143 healthy controls	Case–control study	rs2070744 rs1799983 rs869109213
Diler et al. (2016) [45]	Turkey/Turkish	84 PCa patients 116 healthy controls	Case–control study	rs2070744 rs1799983 rs869109213
Polat et al. (2016) [39]	Turkey/Turkish	50 PCa patients 50 healthy controls	Case–control study	rs2070744 rs1799983 rs869109213
Chen et al. (2018) [48]	Taiwan/Taiwanese	139 premenopausal and 144 postmenopausal BC patients 100 premenopausal and 100 postmenopausal healthy women	Case–control study	rs2070744 rs1799983 rs869109213
Huang et al. (2018) [51]	Taiwan/Taiwanese	277 LC patients	Cohort study	rs2070744 rs1799983
Su et al. (2018) [42]	Taiwan/Taiwanese	1044 OSCC patients 1200 healthy controls	Case–control study	rs2070744 rs1799983
Hung et al. (2019) [46]	Taiwan/Taiwanese	117 UCC patients 95 patients with cervical precancerous lesions 330 healthy controls	Case–control study	rs2070744 rs1799983
Nan et al. (2019) [38]	Mixed	41 case–control studies	Meta-analysis	rs2070744 rs1799983 rs869109213
Tsay et al. (2019) [52]	Taiwan/Taiwanese	431 UC patients 862 healthy controls	Case–control study	rs2070744 rs1799983
Abedinzadeh et al. (2020) [40]	Mixed	4464 cancer cases and 4347 controls (rs1799983) 589 cancer cases and 789 controls (rs869109213) 588 cancer cases and 692 controls (rs2070744)	Meta-analysis	rs2070744 rs1799983 rs869109213
Carkic et al. (2020) [57]	Serbia/Serbian	50 OSCC patients 110 healthy controls	Case–control study	rs2070744 rs1799983 rs869109213
Koçer et al. (2020) [56]	Turkey/Turkish	107 LC patients 100 healthy controls	Case–control study	rs1799983 rs869109213
Balci et al. (2023) [44]	Turkey/Unclear	48 PCa patients 42 biopsy individuals 27 healthy controls	Case–control study	rs2070744

Abbreviations: BC, breast cancer; BLCA; bladder cancer; CRC, colorectal cancer; EMCA, endometrial carcinoma; GC, gastric cancer; LC, lung cancer; PCa, prostate cancer; OSCC, oral squamous cell carcinoma; UC, urothelial cell carcinoma; UCC, uterine cervical cancer; USA, United States of America.

## 3. Consequences of ED

As previously mentioned, ET-1, Ang II, and thrombin constitute EDCFs. Impaired vasodilation from ED implies the release and action of these vasoconstrictors inhibiting the anti-inflammatory and anti-coagulant attributes of healthy ECs [6]. Various genetic variations have been documented to influence the expression and/or activity of these molecules during ED. Consequently, investigating the role of these polymorphisms holds promise for elucidating the molecular mechanisms underlying ED and may offer novel therapeutic targets for managing ED and controlling cancer growth and progression (Table 2).

### 3.1. Endothelin-1 (ET-1)

ET-1, a potent vasoconstrictor peptide, is crucial in regulating vascular tone and endothelial function. Genetic variations within the ET-1 gene (*EDN1*) have been suggested to modulate the protein expression and/or activity, impacting vascular homeostasis and predisposing individuals to ED-related pathologies [58,59].

The association between *EDN1* and cancer has been documented with three distinct SNPs: rs5370 (C>A), a missense variation encoding an aspargine (N) instead of a lysine (K) [60]; rs1800541 (T>G), an alteration located at the gene promotor; and rs2070699 (G>T), an intronic variation [59]. Concerning rs5370, the presence of the A allele (forward strand) was associated with papillary thyroid cancer in individuals over 40 years, notably in men [61]. In contrast, the minor alleles of rs1800541 and rs2070699 seem to confer protection regarding osteosarcoma prognosis. Likewise, the rs1800541 G allele was associated with a reduced risk for pulmonary metastasis and chemoresistance. For the latter condition, the rs2070699 T allele was also related to a decreased risk [58,62]. Concordantly, a haplotype with a greater risk for hormone-refractory PCa was established with the ancestral alleles—rs1800541 T and rs2070699 G [63].

### 3.2. Angiotensin II (Ang II)

Ang II plays a pivotal role in vascular homeostasis. It is synthesized from angiotensinogen, encoded by the *AGT* gene, and further processed by the angiotensin-converting enzyme (ACE), encoded by a gene with the same name [64,65,66,67,68]. Variations in these genes may influence Ang II levels, impacting endothelial function.

An indel (I/D) polymorphism in *ACE* intron 16 is extensively documented in the literature. Current evidence indicates that the D allele carriers have increased ACE expression and activity [64,69]. Furthermore, this indel is referred to as influencing the risk of cancer [70,71,72]. Regarding GC, the DD genotype was associated with a higher risk of gastric tumorigenesis, lymph node metastasis and advanced clinical stages [64,69,73,74,75]. However, according to a 2015 meta-analysis, these associations are only observed in population-based studies, as the I allele seems to confer an increased risk to GC in hospital-based studies [76]. The *ACE* Indel is also referred to as contributing to the PCa risk in Latino and Asian ethnic groups [77,78]. Moreover, DD carriers also present more advanced stages of the disease and early-age diagnostics [79,80,81,82,83]. The same genotype seems to be related to an increased risk of oral precancerous lesions in betel quid chewers and OSCC and lymph node metastasis in men [84,85]. In disagreement, the II genotype was also associated with a three-fold risk of OSCC development [86,87,88]. The I allele could also be linked to the occurrence of EMCA (particularly in normotensive women under 63 years old), endometriosis and leiomyomas [67,89]. Regarding CRC, this indel is considered to have a gender-dependent effect. While D male carriers present larger tumours than those with the II genotype, females carrying the DD genotype have higher survival rates when compared to I carriers [90]. Another study showed an increased risk of early relapse and higher TNM stage for I allele carriers [91]. Contrariwise, the D allele correlates with poor differentiation and lymph node metastasis [92,93]. Regarding LC development, the I allele has a negative effect, particularly when combined with smoking habits in the older population [94,95,96]. In opposition, DD carriers have an increased susceptibility to squamous cell carcinoma development and smoking-related cancer death [97,98]. On the other hand, compared to the other genotypes, heterozygous individuals are suggested to have a raised non-small cell lung cancer (NSCLC) predisposition [99]. Moreover, the ID genotype seems to be related to adrenal incidentalomas compared to the controls [100]. Additionally, II and DD genotypes confer susceptibility to pancreatic cancer (PC) and chronic pancreatitis, respectively [101]. Regarding BC, the I allele carriers show a decreased risk [66,102,103,104,105]. Those with the I allele have a greater expression of HER2 [106], while DD genotype carriers present a better disease-free survival rate [106,107]. On the contrary, the DD genotype seems to be concomitant with worse prognostic factors in premenopausal women and decreased cancer-free survival in postmenopausal women [108,109,110,111]. Regarding hepatocellular carcinoma (HCC) progress, two different studies demonstrated conflicting results, showing decreased and increased risk for DD carriers, respectively [112,113]. Additionally, the ID genotype is also suggested to exert a protective role against BC [114]. The D allele presence is associated with an increased risk of uterine leiomyoma [115]; gall bladder carcinoma (GBC) [116], and glioma [117], while the homozygous D genotype is associated with increased susceptibility to glioma development and low overall survival [118,119]; renal cell carcinoma (RCC) [120]; BLCA [121]; basal cell carcinoma (BCC) [122,123,124]; poor leukaemia survival rates [125]; lymph nodes metastasis in laryngeal cancer (LaC) [126]; pituitary adenomas development and progression [127]; and EMCA [128]. Regarding cancer patients’ prognosis, while a direct impact is not described, the *ACE* ID genotype was associated with higher haemoglobin levels and overall lower fat mass and muscle strength in patients at advanced stages compared to the II genotype [129].

The *ACE* rs4291 (T>A) is an alteration in the promoter region referred to confer susceptibility to cancer in the Asian and Caucasian ethnic groups and specifically to BC in Latino populations [68,103]. This SNP seems to be in linkage disequilibrium (LD) with the *ACE* indel among women. Those with the low-activity alleles (A and I of each polymorphism, respectively) showed decreased BC risk [66]. Moreover, women present a greater risk of BC when carrying the *ACE* rs4291 T allele and rs4343 (G>A) G allele concurrently [130].

The D allele carriers aged between 36 to 54 years old are reported to present a greater risk of BC, whereas a reduced risk was associated with the II/AG and II/CC of *ACE* indel/*AGT* rs699 (A>G) and *ACE* indel/*AGT* rs4762 (G>A) haplotypes, respectively [131].

The *AGT* rs699 and rs4762 are two missense variants. The first one implicates a replacement of methionine by threonine in exon 2, whereas rs4762 represents a substitution of threonine with methionine at position 174 in the amino acid sequence [130]. Furthermore, nodal spread in intestinal-type GC correlates with the combined expression of this Indel and angiotensin II receptor type I (AT1R) [132]. Also, regarding *Helicobacter pylori* (HP) status, negative individuals seem to present a decreased risk of GC [133], whereas, in the HP-positive group with atrophy, the ID genotype seems to confer an increased risk [134].

The G allele of *AGT* rs699 was suggested to be associated with an increased risk of BCC [135], BLCA [136] and CRC [137]. However, in a 2023 study of the same population, the heterozygous genotype was significantly more frequent in the BCC patient group than in the controls [122]. The AA genotype was associated with decreased disease-free survival of BC [138]. Furthermore, the rs699 in the *AGT* gene showed reduced prevalence in Australian EMCA women [65]. Moreover, several *AGT* SNPs, namely rs7539020 (C>T), rs3889728 (C>G), rs3789662 (A>G), rs1326889 (C>T), and rs2493137 (T>C), are suggested to modulate renal cell cancer susceptibility among hypertensive or overweight individuals [139]. Regarding CRC, a greater prevalence of the AG/AG haplotype for rs699/rs5051 (C>T) was found in men [140].

### 3.3. Thrombin

Thrombin, a serine protease originating from prothrombin (its inactive precursor, encoded by *coagulation factor 2* (*F2*)), is a key player in haemostasis, coordinating platelet aggregation and blood coagulation. Its impact extends to diverse cellular functions, including chemotaxis, proliferation, extracellular matrix remodelling, and cytokine release. Just as Factor V Leiden, *F2* rs1799963 (G>A) is well-established as a risk factor for VTE [141,142]. This SNP is located at nucleotide position 20210 within the promoter region. The A allele leads to elevated levels of prothrombin, consequently increasing thrombin generation and favouring thrombogenesis [3,143]. Female carriers of the *F2* rs1799963 A allele with gynaecological malignancies are suggested to show advanced cancer stages at the time of surgery [144]. The rs1799963 AG genotype was also associated with a five-fold increased risk for HCC in subjects with *hepacivirus* [145]. Regarding CRC, whereas an increased susceptibility was correlated with the AA genotype, the AG genotype presented 30% less predisposition for its development [146,147].

**Table 2:** Epidemiological studies on the role of polymorphisms in vasoconstrictors-encoding genes on cancer susceptibility and progression.

First Author (Year)	Country/Ethnic Background	Population Characteristics	Study Design	Studied Polymorphisms
Hajek et al. (2003) [125]	Czech Republic/ Unclear	25 leukaemia patients	Cohort study	*ACE* indel
Koh et al. (2003) [66]	Singapore/Singaporean	189 BC patients 671 healthy controls	Nested case–control study	*ACE* indel *ACE* rs4291
Tormene et al. (2003) [144]	Italy/Unclear	52 women operated for gynaecological malignancy 198 women operated for gynaecological non-malignant disease	Case–control study	*F2* rs1799963
Freitas-Silva et al. (2004) [67]	Portugal/Portuguese	70 EMCA patients 101 healthy controls	Case–control study	*ACE* indel
Medeiros et al. (2004) [80]	Portugal/Portuguese	170 PCa patients 30 healthy controls	Case–control study	*ACE* indel
Chung et al. (2005) [84]	Taiwan/Taiwanese	61 OPL betel quid chewers 61 asymptomatic betel quid chewers	Case–control study	*ACE* indel
Ebert et al. (2005) [73]	Germany/Caucasian	88 GC patients 145 healthy controls	Case–control study	*ACE* indel
González-Zuloeta Ladd et al. (2005) [111]	Netherland/Unclear	4878 female postmenopausal total participants 114 BC patients	Cohort study	*ACE* indel
Goto et al. (2005) [134]	Japan/Japanese	454 GC patients 202 healthy controls	Case–control study	*ACE* indel
Röcken et al. (2005) [75]	Germany/Unclear	113 GC patients 189 healthy controls	Case–control study	*ACE* indel
Arima et al. (2006) [98]	Japan/Japanese	937 total participants 176 subjects died of malignant neoplasm	Cohort study	*ACE* indel
Yaren et al. (2006) [109]	Turkey/Turkish	44 BC patients 46 healthy premenopausal women	Case–control study	*ACE* indel
Carl-McGrath et al. (2007) [69]	Germany/Unclear	45 GC patients	Cohort study	*ACE* indel
González-Zuloeta Ladd et (2007) [138]	Netherlands/Unclear	203 BC cases 3323 controls	Case–control study	*AGT* rs699
Hsieh et al. (2007) [89]	Taiwan/Taiwanese	120 UL patients 125 endometriosis patients 128 healthy controls	Case–control study	*ACE* indel
Röcken et al. (2007) [132]	Germany/Unclear	100 GC patients	Cohort study	*ACE* indel
Röcken et al. (2007) [90]	Germany/Unclear	141 CRC patients 189 healthy controls	Case–control study	*ACE* indel
Vairaktaris et al. (2007) [86]	Greece/Greek and German	60 OSCC patients 153 healthy controls	Case–control study	*ACE* indel
Yaren et al. (2007) [108]	Turkey/Turkish	57 BC patients 52 healthy controls	Case–control study	*ACE* indel
Yigit et al. (2007) [83]	Turkey/Turkish	48 PCa patients 51 healthy controls	Case–control study	*ACE* indel
van der Knaap et al. (2008) [110]	Netherland/Unclear	7679 participants *	Cohort study	*ACE* indel
Alves Corrêa et al. (2009) [114]	Brazil/Brazilian	101 BC patients 307 healthy controls	Case–control study	*ACE* indel
Harman et al. (2009) [100]	Turkey/Turkish	50 adrenal mass patients 30 healthy controls	Case–control study	*ACE* indel *NOS3* rs1799983
Loh et al. (2009) [71]	Mixed/Asian and Caucasian	203 case–control studies	Meta-analysis	*ACE* indel
Vairaktaris et al. (2009) [88]	Mixed/Greek and German	162 OSCC patients 168 healthy controls	Case–control study	*ACE* indel
Vasků et al. (2009) [140]	Czech Republic/Czech	102 CRC patients 101 healthy controls	Case–control study	*AGT* rs699 *AGT* rs5051
Vigano et al. (2009) [129]	Canada/Unclear	72 GC and NSCLC advanced cancer patients	Cohort study	*ACE* indel
Andreotti et al. (2010) [139]	Mixed	1035 RCC patients 777 controls	Case–control study	*AGT* rs7539020 *AGT* rs3889728 *AGT* rs3789662 *AGT* rs1326889 *AGT* rs2493137
Nacak et al. (2010) [95]	Turkey/Turkish	25 LC patients 165 healthy controls	Case–control study	*ACE* indel
Namazi et al. (2010) [106]	Iran/Iranian	70 BC patients 70 healthy controls	Case–control study	*ACE* indel
Srivastava et al. (2010) [116]	India/North Indian	233 GBC patients 260 non-cancer controls	Case–control study	*ACE* indel
Liu et al. (2011) [92]	China/Chinese	241 CRC patients 299 non-cancer controls	Case–control study	*ACE* indel
Lukic et al. (2011) [101]	Serbia/Unclear	45 PC patients 55 chronic pancreatitis patients 128 healthy controls	Case–control study	*ACE* indel
De Martino et al. (2011) [120]	Austria/Unclear	10 RCC patients 173 healthy controls	Case–control study	*ACE* indel
Mendizábal-Ruiz et al. (2011) [104]	Mexico/Mexican	65 BC patients 40 benign breast disease patients	Case–control study	*ACE* indel *AGT* rs699
Vossen et al. (2011) [147]	Germany/German	1801 CRC patients 1853 healthy controls	Case–control study	*F2* rs1799963
Dević Pavlić et al. (2012) [97]	Croatia/Croatian	308 LC patients 353 healthy controls	Case–control study	*ACE* indel
Correa-Noronha et al. (2012) [128]	Brazil/Brazilian	74 EMCA patients and 228 controls 83 EOC patients and 297 controls	Case–control study	*ACE* indel
Huhn et al. (2012) [137]	Mixed/Czech and German	1025 Czech cancer cases and 787 Czech controls 1798 German cancer cases and 1810 German controls	Case–control study	*AGT* rs699
Liu et al. (2012) [85]	Taiwan/Taiwanese	205 male oral cancer patients 88 Oral precancerous lesions patients 120 healthy controls	Case–control study	*ACE* indel
Wang et al. (2012) [79]	China/Han Chinese	189 PCa patients 290 non-cancer controls	Case–control study	*ACE* indel
Altas et al. (2013) [127]	Turkey/Unclear	21 hypophyseal adenoma patients 20 healthy controls	Case–control study	*ACE* indel
Fishchuk et al. (2013) [131]	Ukraine/Ukrainian	131 BC patients 102 healthy women	Case–control study	*ACE* indel *AGT* rs699 *AGT* rs4762
Namazi et al. (2013) [107]	Iran/Iranian	110 BC patients	Prospective study	*ACE* indel
Vylliotis et al. (2013) [87]	Mixed/Greek and German	160 OSCC patients 168 healthy controls	Case–control study	*ACE* indel *F2* rs1799963 *AGT* rs699
Yapijakis et al. (2013) [123]	Greece/Greek	92 BCC patients 103 healthy controls	Case–control study	*ACE* indel
Yuan et al. (2013) [112]	China/Chinese	293 HCC patients 384 healthy controls	Case–control study	*ACE* indel *NOS3* rs869109213
Zang et al. (2013) [62]	China/Han Chinese	260 pulmonary metastatic stage III osteosarcoma patients 260 matched pulmonary metastatic stage IIB osteosarcoma patients	Case–control study	*EDN1* rs1800541 *EDN1* rs2070699 *EDN1* rs5370
Phukan et al. (2014) [94]	India/Northeast Indian	151 LC patients 151 controls	Case–control study	*ACE* indel
Xie et al. (2014) [82]	Mixed	7025 cancer cases 34,911 controls	Meta-analysis	*ACE* indel
Zhang et al. (2014) [70]	Mixed	5007 cancer cases 8173 controls	Meta-analysis	*ACE* indel
Zhou et al. (2014) [58]	China/Han Chinese	350 Paediatric osteosarcoma patients with <90% tumour necrosis 350 matched osteosarcoma patients with ≥90% tumour necrosis	Case–control study	*EDN1* rs1800541 *EDN1* rs2070699 *EDN1* rs5370
Ding et al. (2015) [130]	China/Han Chinese	606 BC patients 633 healthy controls	Case–control study	*ACE* rs4291 *ACE* rs4343
Gan et al. (2015) [133]	Mixed/Asian and Caucasian	1480 GC cases 3773 non-cancer controls	Meta-analysis	*ACE* indel
Lian et al. (2015) [119]	China/Chinese	800 glioma patients 800 healthy controls	Case–control study	*ACE* indel
Pabalan et al. (2015) [74]	Mixed	1459 cancer cases 2581 controls	Meta-analysis	*ACE* indel
Wei et al. (2015) [64]	Mixed	1392 cancer cases 2951 controls	Meta-analysis	*ACE* indel
Yang et al. (2015) [76]	Mixed/Asian and White	2903 GC cases 10,833 controls	Meta-analysis	*ACE* indel
Zha et al. (2015) [113]	China/Dai Chinese	210 HCC patients 206 healthy controls	Case–control study	*ACE* indel
Hanafy et al. (2016) [145]	Egypt/Egyptian	280 HCV-infected patients 100 healthy controls	Case–control study	*F2* rs1799963
Pringle et al. (2016) [65]	Australia/Mixed	184 type 1 endometrioid cancer women 153 healthy controls	Case–control study	*AGT* rs699 *ACE* rs4291
Ali et al. (2017) [121]	Pakistan/Pakistani	200 BLCA patients 200 healthy controls	Case–control study	*ACE* indel
Baghad et al. (2017) [146]	Morocco/Moroccan	76 CRC patients 182 healthy controls	Case–control study	*F2* rs1799963
Marques et al. (2017) [91]	Brazil/Admixed Brazilian	140 CRC patients 140 non-cancer controls	Case–control study	*ACE* indel
Xu et al. (2017) [63]	China/Han Chinese	234 PCa patients with HRPC within six years after androgen deprivation therapy 234 matched PCa patients without HRPC within six years after androgen deprivation therapy	Case–control study	*EDN1* rs1800541 *EDN1* rs2070699 *EDN1* rs5370
Zheng et al. (2017) [93]	China/Chinese	146 CRC patients 106 healthy controls	Case–control study	*ACE* indel
Moghimi et al. (2018) [102]	Mixed	2846 BC cases 9299 controls	Meta-analysis	*ACE* indel
Pandith et al. (2018) [118]	India/Indian	12 glioma patients 141 non-cancer controls	Case–control study	*ACE* indel
Peddireddy et al. (2018) [99]	India/South Indian	246 NSCLC patients 250 healthy controls	Case–control study	*ACE* indel *NOS3* rs869109213
Singh et al. (2018) [105]	India/North Indian	161 BC patients 152 healthy women	Case–control study	*ACE* indel
Wang et al. (2018) [77]	Mixed	1098 PCa cases 12,960 controls	Meta-analysis	*ACE* indel
Aydin et al. (2019) [61]	Turkey/Unclear	113 PTC patients 185 healthy controls	Case–control study	*EDN1* rs1800541 *EDN1* rs5370
Benenemissi et al. (2019) [117]	Algeria/Algerian	36 glioma patients 195 healthy controls	Case–control study	*ACE* indel
Keshavarzi et al. (2019) [115]	Iran/Iranian	202 UL patients 211 healthy controls	Case–control study	*ACE* indel
Papaggelopoulos et al. (2019) [135]	Greece/Greek	190 BCC patients 99 healthy controls	Case–control study	*AGT* rs699
Xiao et al. (2019) [68]	Mixed	8 case–control studies	Meta-analysis	*ACE* rs4291
Banerjee et al. (2021) [96]	India/North Indian	154 LC patients 205 healthy controls	Case–control study	*ACE* indel
Dastgheib et al. (2021) [103]	Mixed	35 case–control studies	Meta-analysis	*ACE* indel *ACE* rs4291
Koronellos et al. (2021) [124]	Greece/Greek	104 BCC patients 111 healthy controls	Case–control study	*ACE* indel
Samara et al. (2021) [136]	Greece/Caucasian	73 BLCA patients 73 healthy controls	Case–control study	*AGT* rs699
Du et al. (2022) [81]	Mixed	817 PCa patients 917 controls	Meta-analysis	*ACE* indel
Said et al. (2022) [78]	Tunisia/Tunisian	124 PCa patients 143 healthy controls	Case–control study	*ACE* indel
Kumbul et al. (2023) [126]	Turkey/Unclear	44 LaC patients 61 healthy controls	Case–control study	*ACE* indel
Yapijakis et al. (2023) [122]	Greece/Greek	100 BCC patients 103 healthy controls	Case–control study	*AGT* rs699 *ACE* indel

* Cancer specification not available. Abbreviations: BC, breast cancer; BCC, basal cell carcinoma; BLCA, bladder cancer; CRC, colorectal cancer; EMCA, endometrial cancer; EOC, epithelial ovarian cancer; GBC, gall bladder cancer; GC, gastric cancer; HCC, hepatocellular carcinoma; HCV, hepatitis C virus; HRPC, hormone-refractory prostate cancer; LaC, laryngeal cancer; LC, lung cancer; NSCLC, non-small cell lung cancer; OSCC, oral squamous cell carcinoma; OPL, oral precancerous lesions; PC, pancreatic cancer; PCa, prostate cancer; PTC, papillary thyroid cancer; RCC, Renal Cell Carcinoma; UL, uterine leiomyoma.

## 4. Adhesion Molecules

The ability of ECs to induce vasodilation mediated by NO is the most common way to measure endothelial function using the flow-mediated vasodilation (FMD) test. However, this ultrasound imaging-based test has poor reproducibility due to operational and patient cardiac function variability [6,8,148]. In this context, the use of ED-circulating biomarkers may present a more reliable alternative [7,26].

In addition to endothelial permeability, decreased NO bioavailability induces the expression of important adhesion molecules, namely P-selectin, E-selectin, vWF, ICAM-1 and VCAM-1, which facilitate cell-to-cell interaction, promoting the migration and adhesion of leucocytes [149]. These molecules are indicative of the pro-thrombotic environment that precedes the development of cardiovascular conditions, with the extent of ED serving as a valuable prognostic indicator [7,8,26]. These well-characterized markers can be measured in circulation with readily available commercial immunoassays, exceeding at least four indicative criteria of an ideal marker/test [149]. Specifically, they demonstrate ease of use, cost-effectiveness, operator independence, and superior reproducibility. Nevertheless, as not all markers exhibit high sensitivity, combining various methodologies, such as microparticles and CECs, would be advocated [150]. 

Given the implications of ED in cancer pathways, exploring genetic polymorphisms within genes encoding for adhesion molecules takes on paramount significance (Table 3).

### 4.1. P-Selectin

P-selectin is a product of *SELP*, and a member of the selectin protein family found on the outer membrane of activated ECs. The crucial function of P-selectin in facilitating leukocyte recruitment to the site of inflammation has been proposed as a driver of tumour aggressiveness and a contributing factor to the onset of cancer cachexia [151]. Two *SELP* SNPs are highlighted in this context: the intergenic variant rs3917647 (G>A) and the missense SNP rs6136 (T>G). Regarding the former, the GG and AA genotypes were linked to high and low P-selectin plasma levels, respectively. The unfavourable nature of the rs3917647 GG genotype in patients with head and neck cancer (HNC) suggests this alternation as a protective factor against cancer malnutrition and potential cachexia [151]. Likewise, the rs6136 T allele was linked to increased expression of *SELP* mRNA, while the G allele was associated with reduced serum P-selectin levels. The evidence pinpoints the rs6136 T allele as a protective factor for cancer cachexia in HNC patients, as well [151,152]. These findings were concordant in locally advanced and metastatic PC [153].

### 4.2. E-Selectin

E-selectin, encoded by *SELE*, is another significant member of the selectin family. This protein plays a fundamental role in promoting tumour angiogenesis and cancer progression, facilitating interactions between cancer cells and endothelial monolayers, especially during the metastatic process, underlining its importance in early metastasis stages [154]. Concordantly, some tumours, particularly BC and CRC, were found to express E-selectin ligands [155,156].

Four SNPs of *SELE* are described in the literature. Starting with rs5361 (T>G), this missense polymorphism causes the exchange of an uncharged serine with a positively charged arginine within the epidermal growth factor domain. This alteration possesses the capability to alter ligand affinity [156]. The ancestral T allele was demonstrated to be a protective factor against BC [157,158]. The negative impact of the SNP G allele is confirmed among GC patients, with the allele being associated with disease development and poor prognosis [159,160]. The same allele was also associated with an increased risk of PC and ovarian cancer (OC) and a worsened prognosis for BC patients [161,162,163]. Likewise, this allele was also linked to an elevated risk of relapse, metastasis and mortality among CRC patients [154,155,156]. A meta-analysis suggested rs5361 as an overall cancer risk factor among Caucasian and Asian ethnic groups [164]. In the same fashion as rs5361, rs5362 (A>G), rs5367 (A>G), and rs5368 (G>A) variant alleles were demonstrated to be associated with an increased risk of BC. Among them, only rs5368 causes a change in the amino acid sequence from histidine to tyrosine [154].

### 4.3. Von Willebrand Factor (vWF)

Besides P-selectin, vWF emerges as one of the molecules meeting the criteria for robust biomarkers of ED [148]. Only one *VWF* polymorphism is identified to influence cancer pathways, namely the intronic SNP rs73049469 (C>A). The variant A allele is linked to lower *VWF* expression at the transcription levels, and it is shown to be associated with worse overall survival among NSCLC patients [165,166].

### 4.4. ICAM-1

This cell adhesion molecule, a member of the Ig-superfamily, serves as a crucial factor in the recruitment, activation, and facilitation of leukocyte functions at inflammatory sites. As a result of proteolytic cleavage, its soluble form becomes notably elevated in both inflammatory and malignant conditions [167,168]. Six polymorphisms within *ICAM1* have been associated with tumorigenic roles: rs5498 (A>G), rs1799969 (G>A), rs281437(C>T), rs1437 (A>G), rs923366 (C>T) and rs3093030 (C>T).

The rs5498 polymorphism represents a missense variant within exon 6, giving rise to an amino acid substitution from glutamine (E) to lysine (K). This shift affects the splicing of *ICAM1* mRNA, leading to a higher concentration of the soluble protein [168,169,170]. Beyond its well-established association with atherosclerosis, this SNP has garnered attention for its diverse implications in various cancer types. However, its effects remain the subject of debate, as it can either confer risk or protection depending on the tumour [171]. Namely, the homozygous minor allele (G allele) genotype was associated with an increased risk of cancer in the Asian ethnic group but decreased risk in Europeans [172,173]. The G allele was also related to an increased risk of OSCC but diminished for CRC and melanoma [168,173,174]. Furthermore, this allele seems to increase the susceptibility to CRC, especially for older individuals [175,176,177,178]. When in homozygosity, the presence of the lysine correlates with well-differentiated CRC [179]. The G variant allele is also suggested to be a protective factor for cervical adenocarcinoma [169]. In GC, the AA genotype was associated with an augmented risk and a higher likelihood of metastasis compared to the G allele [168,180]. Likewise, the A allele showed an association with advanced stages and poorer survival rates among NSCLC patients [181]. In opposition, the G allele was related to the risk of OC (especially for those with first-degree hereditary tumours or precocious menarche), UC development and invasive stages, HCC in smokers, PCa development, precancerous lesions in uterine cervical carcinogenesis and gliomas development [182,183,184,185,186,187].

The rs1799969 SNP results in an exchange of a glycine for an arginine in exon 4, at codon 241, with the ability to alter the functional activity of ICAM-1 and consequently grant the capacity to recruit and activate immune cells. The variant A allele was shown to be associated with higher cancer risk [172,188]. The presence of the A allele was linked to gliomas and CRC and the GA genotype to BC [168,177]. The A/G haplotype for rs1799969/rs5498 is associated with an increased risk of BC, while it is suggested to exert a protective effect on primary brain tumours [167,168].

The variant T alleles of rs281437 and rs923366 *ICAM1* SNPs, two 3′ UTR variants, were associated with increased and reduced risk of primary HCC, respectively [189]. Nonetheless, the CC genotype of rs281437 seems to be related to a higher risk of BC development when compared to the other genotypes [190]. As for rs1437, a 3′ UTR located SNP, the variant G allele was linked to OC augmented risk [191].

Although the functional consequence of rs3093030 is unknown, a protective effect of the variant T allele was found for UCC and primary HCC [169,189]. In contrast, in a different population, women seem to be more susceptible to invasive uterine cervical carcinogenesis when the variant allele is in homozygosity. The C/G, T/A and T/G haplotypes of rs3093030/rs5498 were shown to increase the risk of precancerous lesions and invasive UCC [186]. For UCC, a reduced risk C/T/G haplotype of rs281432(G>C)/rs3093030/rs5498 was discovered [169].

### 4.5. VCAM-1

Similarly to ICAM-1, VCAM-1 acts in the immune-endothelial communication system, contributing to inflammatory and immune processes and cancer metastasis [192]. Numerous *VCAM1* polymorphisms have been linked to cancer. The intronic variation rs3176861 (C>T) currently has an unknown functional consequence. Nevertheless, the presence of the T allele relates to a substantial decrease in the odds of developing lymphedema after BC surgery [193]. The polymorphism rs1041163 (T>C) is an intergenic variant located within exon 9. The SNP C allele was deemed a protective factor for non-Hodgkin lymphoma (NHL) [194]. In opposition, for the synonymous variant rs3176879 (G>A), the variant allele seems to confer susceptibility to recurrent BLCA in patients submitted to immunotherapy [195,196].

**Table 3:** Epidemiological studies on the role of ED-related adhesion molecules gene polymorphisms on cancer susceptibility and progression.

First Author (Year)	Country/Ethnic Background	Population Characteristics	Study Design	Studied Polymorphisms
Chen et al. (2006) [185]	USA/African-American	286 PCa patients 391 healthy controls	Case–control study	*ICAM1* rs5498
Theodoropoulos et al. (2006) [177]	Greece/Greek	222 CRC patients 200 healthy controls	Case–control study	*ICAM1* rs5498 *ICAM1* rs1799969
Alessandro et al. (2007) [156]	Italy/Caucasian	172 CRC patients 80 healthy controls	Case–control study	*SELE* rs5361
Arandi et al. (2008) [167]	Iran/southern Iranian	276 BC patients and 235 healthy controls 264 BC patients and 200 healthy controls	Case–control study	*ICAM1* rs1799969 *ICAM1* rs5498
Burim et al. (2009) [187]	Brazil/Unclear	158 astrocytoma patients and 162 controls	Case–control study	*ICAM1* rs5498 *ICAM1* rs1799969
Wang et al. (2009) [179]	China/Chinese	87 CRC patients 102 non-CRC controls	Case–control study	*ICAM1* rs5498 *ICAM1* rs1799969
Wang et al. (2009) [194]	Jamaica/Jamaican	395 NHL patients 309 non-NHL controls	Case–control study	*VCAM1* rs1041163
Panoussopoulos et al. (2010) [161]	Greece/Unclear	80 PC patients 160 healthy controls	Case–control study	*SELE* rs5361
Naidu et al. (2011) [157]	Malaysia/Malaysian	387 BC patients 252 healthy controls	Case–control study	*SELE* rs5361
Tan et al. (2012) [152]	Scotland and Canada/Unclear	775 cancer patients 101 validation cohort patients	Cohort study	*SELP* rs6136 *ICAM1* rs281432
Thanopoulou et al. (2012) [181]	Greece/Unclear	203 NSCLC patients 175 healthy controls	Case–control study	*ICAM1* rs5498
Tian et al. (2012) [180]	China/Chinese	332 GC patients 380 healthy controls	Case–control study	*ICAM1* rs5498
Xia et al. (2012) [159]	China/Chinese	311 GC patients 425 controls	Case–control study	*SELE* rs5361
Kontogianni et al. (2013) [163]	Greece/Unclear	261 BC patients 480 healthy controls	Case–control study	*SELE* rs5361
Liarmakopoulos et al. (2013) [160]	Greece/Greek	88 GC patients 480 healthy controls	Case–control study	*SELE* rs5361
Lin et al. (2013) [174]	Taiwan/Unclear	595 OSCC patients 561 healthy controls	Case–control study	*ICAM1* rs5498
Miaskowski et al. (2013) [193]	Mixed	155 BC patients with lymphedema 387 BC patients without lymphedema	Case–control study	*VCAM1* rs3176861
Yilmaz et al. (2013) [168]	Turkey/Turkish	92 primary brain tumour patients 92 healthy controls	Case–control study	*ICAM1* rs5498 *ICAM1* rs1799969
Avan et al. (2014) [153]	Italy/Unclear	303 locally advanced or metastatic PC	Cohort study	*SELP* rs6136
Cai et al. (2014) [182]	China/Northern Han Chinese	408 OC patients 520 healthy controls	Case–control study	*ICAM1* rs5498
Cheng et al. (2014) [164]	Mixed/Asian and Caucasian	1675 cancer patients 2285 controls	Meta-analysis	*SELE* rs5361
Wang et al. (2014) [183]	Taiwan/Taiwanese	279 UC patients 279 healthy controls	Case–control study	*ICAM1* rs5498
Andrew et al. (2015) [196]	USA/Caucasian	783 UC patients	Cohort study	*VCAM1* rs3176879
Cheng et al. (2015) [172]	Mixed	4844 cancer patients 5618 healthy controls	Meta-analysis	*ICAM1* rs5498 *ICAM1* rs1799969
Tang et al. (2015) [173]	Mixed	5528 cancer patients and 6173 controls for rs5498 3138 cancer cases and 3699 controls for rs3093030	Meta-analysis	*ICAM1* rs5498 *ICAM1* rs3093030
Chen et al. (2016) [184]	Taiwan/Taiwanese	305 HCC patients 613 healthy controls	Case–control study	*ICAM1* rs5498
Ghazy et al. (2016) [191]	Egypt/Unclear	60 mixed-type OC patients 20 healthy controls	Case–control study	*ICAM1* rs1437
Golnarnik et al. (2016) [158]	Iran/Northern Iranian	100 BC patients 120 healthy controls	Case–control study	*SELE* rs5361
Lu et al. (2016) [162]	China/Chinese	687 OC patients 687 healthy controls	Case–control study	*SELE* rs5361
Novikov et al. (2016) [178]	Russia/unclear	49 CRC patients 30 BC patients 33 controls	Case–control study	*ICAM1* rs5498
Sun et al. (2016) [186]	Taiwan/Taiwanese	91 UCC patients 63 patients with precancerous lesions 290 healthy controls	Case–control study	*ICAM1* rs5498 *ICAM1* rs3093030 *ICAM1* rs281432
Zhang et al. (2016) [188]	Mixed	4608 cancer patients 4913 controls	Meta-analysis	*ICAM1* rs1799969 *ICAM1* rs3093030
Liu et al. (2017) [175]	China/Chinese	195 CRC patients 188 healthy controls	Case–control study	*ICAM1* rs5498
Powrózek et al. (2019) [151]	Poland/Unclear	62 HNC patients	Cohort study	*SELP* rs3917647 *SELP* rs6136
Qian et al. (2019) [166]	Mixed European/Caucasian	948 NSCLC patients	Cohort study	*VWF* rs73049469
Ghazy et al. (2020) [190]	Egypt/Egyptian	40 BC patients 40 healthy controls	Case–control study	*ICAM1* rs281437
Feng et al. (2021) [169]	China/Northern Chinese Han	488 UCC patients 684 patients with cervical precancerous lesions 510 healthy females	Case–control study	*ICAM1* rs5498 *ICAM1* rs3093030 *ICAM1* rs281432
He et al. (2021) [189]	China/Unclear	290 HCC patients 290 healthy controls	Case–control study	*ICAM1* rs281437 *ICAM1* rs923366 *ICAM1* rs3093030
Qiu et al. (2021) [176]	Mixed	1003 CRC patients 1303 healthy controls	Case–control study	*ICAM1* rs5498 *ICAM1* rs3093030
Zakariya et al. (2022) [154]	Iraq/Iraqi	60 BC patients 40 healthy controls	Case–control study	*SELE* rs5361 *SELE* rs5368 *SELE* rs5362

Abbreviations: BC, breast cancer; CRC, colorectal cancer; GC, gastric cancer; HCC, hepatocellular carcinoma; HNC, head and neck cancer; OC, ovarian cancer; OSCC, oral squamous cell carcinoma; PCa, prostate cancer; PC, pancreatic cancer; NHL, non-Hodgkin lymphoma; NSCLC, non-small cell lung carcinoma; UC, urothelial cell carcinoma; UCC, uterine cervical cancer.

## 5. ED-Related Proteins and Cancer Hallmarks

Overall, despite data inconsistencies, ED-related genetic polymorphisms appear to impact the tumorigenic process. Literature suggests a complex relationship between ED and cancer, with the former playing a multifaceted role in the risk and progression of the latter [11,197,198]. By examining the specific contributions of proteins associated with ED to different hallmarks of cancer, the molecular mechanisms underlying tumour formation and dissemination can be further dissected. This knowledge lays the groundwork for validating the role of ED-related genetic polymorphisms (Table 4) in cancer biology, enriching our comprehension of the intricate interplay between the two conditions.

**Figure 3:**
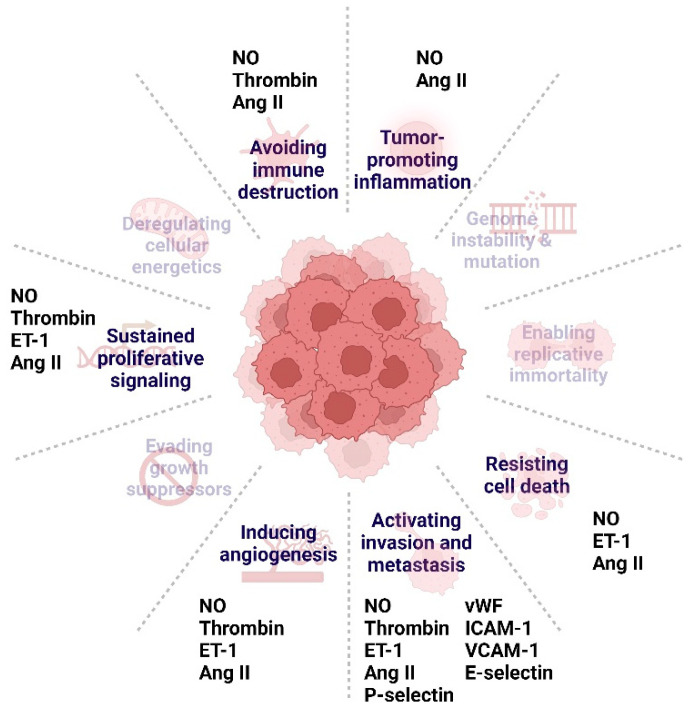
Endothelial dysfunction-related proteins and their respective contribution to cancer hallmarks. Hallmarks depicted with transparency denote the absence of documented endothelial dysfunction-related proteins contributing to them. Abbreviations: Ang II, Angiotensin II; ET-1, Endothelin-1; ICAM-1, intercellular adhesion molecule-1; NO, nitric oxide; VCAM-1, vascular cell adhesion molecule-1; vWF, von Willebrand factor. Figure created with Biorender.com (accessed on 13 April 2024).

From a protein standpoint, as already discussed, NO can exert a dual role in cancer, modulating cell proliferation and apoptosis in a concentration-dependent manner, with low concentrations promoting cell proliferation and anti-apoptotic responses and vice versa [12]. Furthermore, NO dysregulation can foster a pro-thrombotic and pro-inflammatory environment, which promotes tumour proliferation, limits immune response and facilitates angiogenesis and metastasis [199]. Impaired vasodilation raises the action of vasoconstrictors, such as ET-1, Ang II and thrombin. Besides thrombosis, these molecules play a role in tumorigenesis by promoting cellular proliferation, angiogenesis, and metastasis. Regarding ET-1, it triggers sustained proliferative signalling, apoptosis evasion, and migration and invasion, through its receptor ET_A_ [61,200]. Additionally, it promotes angiogenesis by fibroblast stimulation, resulting in remodelling and deposition of the extracellular matrix (ECM) and consequent release of angiogenic factors [200]. Similarly to ET-1, Ang II is a mitogenic and pro-angiogenic vasoconstrictor that promotes tumour angiogenesis and inflammation through the upregulation of vascular endothelial growth factor (VEGF) and prostaglandins [201]. Moreover, upon binding to its receptors, AT1R and AT2R, Ang II activates signalling pathways of cell proliferation. Interestingly, AT1R (unlike AT2R) exhibits anti-apoptotic properties [202,203]. Lastly, thrombin can stimulate DNA synthesis and upregulate several growth and angiogenesis-related genes by activating the protease-activated receptor 1 (PAR-1) pathway [204,205]. Furthermore, by promoting the overexpression of adhesion molecules, these vasoconstrictors may facilitate immune evasion and tumour invasion and metastasis [206,207]. Indeed, the levels of selectins and CAMs in the serum of cancer patients correlate with tumour dissemination [208]. Furthermore, vWF, in combination with thrombin, contributes to the formation of tumour-platelet aggregates, enabling tumour cell survival and their successful metastasis [198]. In summary, proteins associated with ED play a pivotal role in cancer initiation and progression, contributing to various hallmarks of the disease (Figure 3). Thus, polymorphisms within their coding genes may contribute to alterations in cancer susceptibility and progression in patients carrying these variants. Understanding the impact of these DNA variations might enhance our comprehension of cancer development and open avenues for targeted interventions to disrupt these pathways and hinder disease development and progression.

**Table 4:** Characterization of ED-related Genetic Polymorphisms via Ensembl.

Gene	Polymorphism	Substitution	Ancestral Allele	Global MAF (MA)	Most Severe Consequence
*NOS3*	rs2070744	C>G	C	23% (C)	Intron variant
	rs1799983	T>G/A	G	18% (T)	Missense variant
	rs869109213	VNTR	NA	NA	Intron variant
*EDN1*	rs5370	G>T	G	25% (T)	Missense variant
	rs1800541	T>G	T	28% (G)	Regulatory region variant
	rs2070699	G>C/T	G	36% (T)	Intron variant
*ACE*	Indel	Indel	NA	NA	-
	rs4291	T>A/G	A	35% (T)	Regulatory region variant
	rs4343	G>A	A	36% (G)	Synonymous variant
*AGT*	rs699	A>G	G	29% (A)	Missense variant
	rs4762	G>A	G	10% (A)	Missense variant
	rs1326889	C>T/A	T	22% (C)	Intron variant
	rs281432	C>G	G	48% (C)	Intron variant
	rs2493137	T>C	T	48% (T)	Intron variant
	rs5050	T>C/G	G	18% (G)	5 prime UTR variant
	rs5051	C>G/A/T	T	29%(C)	5 prime UTR variant
	rs7539020	C>T	C	49%(C)	Intron variant
	rs3889728	C>G/T	C	30%(T)	Intron variant
	rs3789662	A>G	A	34%(G)	3 prime UTR variant
*F2*	rs1799963	G>A	G	<1% (A)	3 prime UTR variant
*SELP*	rs3917647	G>A	G	46% (A)	Intergenic variant
	rs6136	T>C/G	T	4% (G)	Missense variant
*SELE*	rs5361	T>G/A	T	5% (G)	Missense variant
	rs5362	A>G	G	5% (G)	Non-coding transcript exon variant
	rs5367	A>G	A	5% (G)	Splice region variant
	rs5368	G>A	G	15% (A)	Missense variant
*VWF*	rs73049469	C>A	C	13% (A)	Intron variant
*ICAM1*	rs1437	A>G/T	G	37% (G)	3 prime UTR variant
	rs5498	A>G	A	36% (G)	Missense variant
	rs1799969	G>A	G	6% (A)	Missense variant
	rs281437	C>G/T	C	26% (T)	3 prime UTR variant
	rs923366	C>T/A	T	35% (T)	3 prime UTR variant
	rs3093030	C>T	C	32% (T)	Non-coding transcript exon variant
*VCAM1*	rs3176861	C>T	C	20% (T)	Intron variant
	rs3176879	G>A	A	13% (G)	Synonymous variant
	rs1041163	T>C	T	18% (C)	Intergenic variant

Abbreviations: MA, minor allele; MAF, minor allele frequency; NA, no data available; VNTR, variable number tandem repeats.

## 6. Conclusions

In this comprehensive narrative review, genetic polymorphisms implicated in ED were evaluated for their impact on cancer susceptibility and progression among distinct ethnic groups. Briefly, our examination reveals a tendency for BC as a primary focus in studies concerning multiple ED-related genetic polymorphisms, closely followed by CRC. Notably, BC has garnered widespread attention across various countries, particularly in China, where research efforts have been particularly pronounced. China also stands out for its extensive study of distinct SNPs, a trend also observed in Turkey. Moreover, among the polymorphisms examined, the *ACE* indel distinguishes itself as a frequently studied variant, suggesting its potential relevance in the tumorigenic process. The polymorphisms under study exhibit a clear tendency to modulate cancer risk. The *ACE* indel stands out with over 50 risk associations for cancer, especially for BC, followed by PCa. Across all cancer models, the D allele commonly emerges associated with risk, while inversely the I allele is reported to confer protection. Additionally, as many risk associations for CRC were found for *ICAM1* rs5498 as for the *ACE* indel, despite being much less studied in the general population. Controversy surrounds this SNP, with the G allele being the most frequently associated with cancer risk and also the most frequently associated with protection. Protection against osteosarcoma was solely associated with *EDN1* SNPs, while PCa was mainly studied in relation to *NOS3* SNPs. Overall, BC, PCa, and CRC were the main tumour models in studies concerning ED-related genetic polymorphisms. Most of the variants seem to have a context-dependent role varying upon specific tumour and patient characteristics. It should be noted that many of the conducted studies exhibited significant flaws, such as failing to specify the risk/protection genotype, or not confirming the results with subsequent validation studies. Hence, future studies with larger sample sizes are warranted to elucidate these complexities. Since proteins associated with ED contribute to several hallmarks of cancer, a better understanding of these DNA variations holds promise for the development of precision medicine approaches to improve cancer patient care and enhance clinical outcomes. Inclusively, as a wide range of molecules play relevant roles in ED, the implications of other downstream proteins in tumorigenesis should be dissected. Likewise, given the central role of ED in CAT, the influence of the studied polymorphisms in CAT pathogenesis needs to be clarified.

## Data Availability

The data presented in this study are available on request from the corresponding author.

## References

[1] Marques I.S., Tavares V., Neto B.V., Mota I.N.R., Pereira D., Medeiros R. (2023). Long Non-Coding RNAs in Venous Thromboembolism: Where Do We Stand?. Int. J. Mol. Sci..

[2] Lutsey P.L., Zakai N.A. (2023). Epidemiology and prevention of venous thromboembolism. Nat. Rev. Cardiol..

[3] Tavares V., Neto B.V., Vilas-Boas M.I., Pereira D., Medeiros R. (2022). Impact of hereditary thrombophilia on cancer-associated thrombosis, tumour susceptibility and progression: A review of existing evidence. Biochim. Biophys. Acta Rev. Cancer.

[4] Fernandes C.J., Morinaga L.T.K., Alves J.L.J., Castro M.A., Calderaro D., Jardim C.V.P., Souza R. (2019). Cancer-associated thrombosis: The when, how and why. Eur. Respir. Rev..

[5] Kacimi S.E.O., Moeinafshar A., Haghighi S.S., Saghazadeh A., Rezaei N. (2022). Venous thromboembolism in cancer and cancer immunotherapy. Crit. Rev. Oncol. Hematol..

[6] Poredos P., Jezovnik M.K. (2018). Endothelial Dysfunction and Venous Thrombosis. Angiology.

[7] Zhang J. (2022). Biomarkers of endothelial activation and dysfunction in cardiovascular diseases. Rev. Cardiovasc. Med..

[8] Migliacci R., Becattini C., Pesavento R., Davi G., Vedovati M.C., Guglielmini G., Falcinelli E., Ciabattoni G., Dalla Valle F., Prandoni P. (2007). Endothelial dysfunction in patients with spontaneous venous thromboembolism. Haematologica.

[9] Rajendran P., Rengarajan T., Thangavel J., Nishigaki Y., Sakthisekaran D., Sethi G., Nishigaki I. (2013). The vascular endothelium and human diseases. Int. J. Biol. Sci..

[10] Cyr A.R., Huckaby L.V., Shiva S.S., Zuckerbraun B.S. (2020). Nitric Oxide and Endothelial Dysfunction. Crit. Care Clin..

[11] Franses J.W., Drosu N.C., Gibson W.J., Chitalia V.C., Edelman E.R. (2013). Dysfunctional endothelial cells directly stimulate cancer inflammation and metastasis. Int. J. Cancer.

[12] Choi J.Y., Lee K.M., Noh D.Y., Ahn S.H., Lee J.E., Han W., Jang I.J., Shin S.G., Yoo K.Y., Hayes R.B. (2006). Genetic polymorphisms of eNOS, hormone receptor status, and survival of breast cancer. Breast Cancer Res. Treat..

[13] Trent R.J. (2012). Molecular Medicine.

[14] Chiarella P., Capone P., Sisto R. (2023). Contribution of Genetic Polymorphisms in Human Health. Int. J. Environ. Res. Public Health.

[15] Leaché A.D., Oaks J.R. (2017). The utility of single nucleotide polymorphism (SNP) data in phylogenetics. Annu. Rev. Ecol. Evol. Syst..

[16] Hovhannisyan G., Harutyunyan T., Aroutiounian R., Liehr T. (2019). DNA copy number variations as markers of mutagenic impact. Int. J. Mol. Sci..

[17] Sehn J.K. (2015). Insertions and deletions (indels). Clinical Genomics.

[18] Gemayel R., Vinces M.D., Legendre M., Verstrepen K.J. (2010). Variable tandem repeats accelerate evolution of coding and regulatory sequences. Annu. Rev. Genet..

[19] Theofilis P., Sagris M., Oikonomou E., Antonopoulos A.S., Siasos G., Tsioufis C., Tousoulis D. (2021). Inflammatory mechanisms contributing to endothelial dysfunction. Biomedicines.

[20] Verhamme P., Hoylaerts M. (2006). The pivotal role of the endothelium in haemostasis and thrombosis. Acta Clin. Belg..

[21] Neubauer K., Zieger B. (2022). Endothelial cells and coagulation. Cell Tissue Res..

[22] Shibata S., Harpel P., Bona C., Fillit H. (1993). Monoclonal antibodies to heparan sulfate inhibit the formation of thrombin-antithrombin III complexes. Clin. Immunol. Immunopathol..

[23] Weiler H., Isermann B. (2003). Thrombomodulin. J. Thromb. Haemost..

[24] Sandset P.M. (1996). Tissue factor pathway inhibitor (TFPI)–an update. Pathophysiol. Haemost. Thromb..

[25] Durand M.J., Gutterman D.D. (2013). Diversity in mechanisms of endothelium-dependent vasodilation in health and disease. Microcirculation.

[26] Incalza M.A., D’Oria R., Natalicchio A., Perrini S., Laviola L., Giorgino F. (2018). Oxidative stress and reactive oxygen species in endothelial dysfunction associated with cardiovascular and metabolic diseases. Vascul Pharmacol..

[27] Tavares V., Pinto R., Assis J., Pereira D., Medeiros R. (2020). Venous thromboembolism GWAS reported genetic makeup and the hallmarks of cancer: Linkage to ovarian tumour behaviour. Biochim. Biophys. Acta-Rev. Cancer.

[28] Endemann D.H., Schiffrin E.L. (2004). Endothelial dysfunction. J. Am. Soc. Nephrol..

[29] Vanhoutte P.M., Shimokawa H., Feletou M., Tang E.H. (2017). Endothelial dysfunction and vascular disease—A 30th anniversary update. Acta Physiol..

[30] Dardi P., Dos Reis Costa D.E.F., Assunção H.C.R., Rossoni L.V. (2022). Venous endothelial function in cardiovascular disease. Biosci. Rep..

[31] Vannini F., Kashfi K., Nath N. (2015). The dual role of iNOS in cancer. Redox Biol..

[32] Napoli C., Paolisso G., Casamassimi A., Al-Omran M., Barbieri M., Sommese L., Infante T., Ignarro L.J. (2013). Effects of nitric oxide on cell proliferation: Novel insights. J. Am. Coll. Cardiol..

[33] Gao X., Wang J., Wang W., Wang M., Zhang J. (2015). eNOS Genetic Polymorphisms and Cancer Risk: A Meta-Analysis and a Case-Control Study of Breast Cancer. Medicine.

[34] Krishnaveni D., Amar Chand B., Shravan Kumar P., Uma Devi M., Ramanna M., Jyothy A., Pratibha N., Balakrishna N., Venkateshwari A. (2015). Association of endothelial nitric oxide synthase gene T-786C promoter polymorphism with gastric cancer. World J. Gastrointest. Oncol..

[35] Wang Q., Sun H., Qi X., Zhou M. (2020). eNOS rs2070744 polymorphism might influence predisposition to hemorrhagic cerebral vascular diseases in East Asians: A meta-analysis. Brain Behav..

[36] Wu X., Wang Z.F., Xu Y., Ren R., Heng B.L., Su Z.X. (2014). Association between three eNOS polymorphisms and cancer risk: A meta-analysis. Asian Pac. J. Cancer Prev..

[37] Zhang L., Chen L.M., Wang M.N., Chen X.J., Li N., Huang Y.D., Chen M. (2014). The G894t, T-786c and 4b/a polymorphisms in Enos gene and cancer risk: A meta-analysis. J. Evid. Based Med..

[38] Nan J., Liu Y., Xu C., Ge D. (2019). Effects of eNOS gene polymorphisms on individual susceptibility to cancer: A meta-analysis. Nitric Oxide.

[39] Polat F., Turaçlar N., Yilmaz M., Bingöl G., Cingilli Vural H. (2016). eNOS gene polymorphisms in paraffin-embedded tissues of prostate cancer patients. Turk. J. Med. Sci..

[40] Abedinzadeh M., Dastgheib S.A., Maleki H., Heiranizadeh N., Zare M., Jafari-Nedooshan J., Kargar S., Neamatzadeh H. (2020). Association of Endothelial Nitric Oxide Synthase Gene Polymorphisms with Susceptibility to Prostate Cancer: A Comprehensive Systematic Review and Meta-Analysis. Urol. J..

[41] Lu J., Wei Q., Bondy M.L., Yu T.K., Li D., Brewster A., Shete S., Sahin A., Meric-Bernstam F., Wang L.E. (2006). Promoter polymorphism (-786t>C) in the endothelial nitric oxide synthase gene is associated with risk of sporadic breast cancer in non-Hispanic white women age younger than 55 years. Cancer.

[42] Su C.W., Chien M.H., Lin C.W., Chen M.K., Chow J.M., Chuang C.Y., Chou C.H., Liu Y.C., Yang S.F. (2018). Associations of genetic variations of the endothelial nitric oxide synthase gene and environmental carcinogens with oral cancer susceptibility and development. Nitric Oxide.

[43] Jang M.J., Jeon Y.J., Kim J.W., Chong S.Y., Hong S.P., Oh D., Cho Y.K., Chung K.W., Kim N.K. (2013). Association of eNOS polymorphisms (-786T>C, 4a4b, 894G>T) with colorectal cancer susceptibility in the Korean population. Gene.

[44] Balci S., Akbayir S., Bozlu M., Tamer L. (2023). Investigation of the relationship between endothelial nitric oxide synthase T786C polymorphism and PSA, PSA derivatives, and prostate cancer in the Turkish population. J. Med. Biochem..

[45] Diler S.B., Öden A. (2016). The T-786C, G894T, and Intron 4 VNTR (4a/b) Polymorphisms of the Endothelial Nitric Oxide Synthase Gene in Prostate Cancer Cases. Genetika.

[46] Hung W.C., Wu T.F., Ng S.C., Lee Y.C., Shen H.P., Yang S.F., Wang P.H. (2019). Involvement of endothelial nitric oxide synthase gene variants in the aggressiveness of uterine cervical cancer. J. Cancer.

[47] Oztürk E., Dikensoy E., Balat O., Uğur M.G., Balcı S.O., Aydın A., Kazancı U., Pehlivan S. (2011). Association of endothelial nitric oxide synthase gene polymorphisms with endometrial carcinoma: A preliminary study. J. Turk. Ger. Gynecol. Assoc..

[48] Chen C.H., Wu S.H., Tseng Y.M., Hou M.F., Tsai L.Y., Tsai S.M. (2018). Distinct role of endothelial nitric oxide synthase gene polymorphisms from menopausal status in the patients with sporadic breast cancer in Taiwan. Nitric Oxide.

[49] Arıkan S., Cacina C., Guler E., Çulcu S., Tuna G., Yaylım-Eraltan I. (2012). The effects of NOS3 Glu298Asp variant on colorectal cancer risk and progression in Turkish population. Mol. Biol. Rep..

[50] Polat F., Diler S.B., Azazi İ., Öden A. (2015). T-786C, G894T, and intron 4 VNTR (4a/b) polymorphisms of the endothelial nitric oxide synthase gene in bladder cancer cases. Asian Pac. J. Cancer Prev..

[51] Huang C.Y., Hsieh M.J., Wu W.J., Chiang W.L., Liu T.C., Yang S.F., Tsao T.C. (2018). Association of endothelial nitric oxide synthase (eNOS) polymorphisms with EGFR-mutated lung adenocarcinoma in Taiwan. J. Cancer.

[52] Tsay M.D., Hsieh M.J., Wang S.S., Wang W.C., Chou Y.Y., Shih C.H., Yang S.F., Chou Y.E. (2019). Impact of endothelial nitric oxide synthase polymorphisms on urothelial cell carcinoma development. Urol. Oncol..

[53] Raina P., Sikka R., Gupta H., Matharoo K., Bali S.K., Singh V., Bhanwer A. (2021). Association of eNOS and MCP-1 Genetic Variants with Type 2 Diabetes and Diabetic Nephropathy Susceptibility: A Case–Control and Meta-Analysis Study. Biochem. Genet..

[54] Yeh C.C., Santella R.M., Hsieh L.L., Sung F.C., Tang R. (2009). An intron 4 VNTR polymorphism of the endothelial nitric oxide synthase gene is associated with early-onset colorectal cancer. Int. J. Cancer.

[55] Ramírez-Patiño R., Figuera L.E., Puebla-Pérez A.M., Delgado-Saucedo J.I., Legazpí-Macias M.M., Mariaud-Schmidt R.P., Ramos-Silva A., Gutiérrez-Hurtado I.A., Gómez Flores-Ramos L., Zúñiga-González G.M. (2013). Intron 4 VNTR (4a/b) polymorphism of the endothelial nitric oxide synthase gene is associated with breast cancer in Mexican women. J. Korean Med. Sci..

[56] Koçer C., Benlier N., Balci S.O., Pehlivan S., Şanli M., Nacak M. (2020). The role of endothelial nitric oxide synthase gene polymorphisms in patients with lung cancer. Clin. Respir. J..

[57] Carkic J., Nikolic N., Nisevic J., Lazarevic M., Kuzmanovic-Pficer J., Jelovac D., Milasin J. (2020). Endothelial nitric oxide synthase polymorphisms/haplotypes are strong modulators of oral cancer risk in Serbian population. J. Oral Sci..

[58] Zhou Y., Liu B., Wang M., Ni J. (2014). Endothelin-1 gene polymorphisms and risk of chemoresistant pediatric osteosarcoma. Pediatr. Blood Cancer.

[59] Ahmed M., Rghigh A. (2016). Polymorphism in Endothelin-1 Gene: An Overview. Curr. Clin. Pharmacol..

[60] Kirshbom P.M., Mahle W.T., Joyner R.W., Leong T., Wilson M., Kogon B.E., Kanter K.R., Bouzyk M.M. (2008). The endothelin-1 G5665T polymorphism impacts transplant-free survival for single ventricle patients. J. Thorac. Cardiovasc. Surg..

[61] Aydin A.F., Vural P., Doğru-Abbasoğlu S., Çil E. (2019). The endothelin 1 and endothelin receptor A gene polymorphisms increase the risk of developing papillary thyroid cancer. Mol. Biol. Rep..

[62] Zang X., Zhou Y., Huang Z., Zhang C. (2013). Endothelin-1 single nucleotide polymorphisms and risk of pulmonary metastatic osteosarcoma. PLoS ONE.

[63] Xu D., Wang X., Lou Y. (2017). Association of endothelin-1 gene single-nucleotide polymorphisms and haplotypes with risk of hormone refractory prostate cancer. Pharmazie.

[64] Wei M.T., Chen N., He Y.Z., Wang J.R., Yang Y., Guo X.J., Wang Z.Q. (2015). Angiotensin-converting enzyme insertion/deletion polymorphism and gastric cancer: A systematic review and meta-analysis. Clin. Res. Hepatol. Gastroenterol..

[65] Pringle K.G., Delforce S.J., Wang Y., Ashton K.A., Proietto A., Otton G., Blackwell C.C., Scott R.J., Lumbers E.R. (2016). Renin-angiotensin system gene polymorphisms and endometrial cancer. Endocr. Connect..

[66] Koh W.P., Yuan J.M., Sun C.L., van den Berg D., Seow A., Lee H.P., Yu M.C. (2003). Angiotensin I-converting enzyme (ACE) gene polymorphism and breast cancer risk among Chinese women in Singapore. Cancer Res..

[67] Freitas-Silva M., Pereira D., Coelho C., Bicho M., Lopes C., Medeiros R. (2004). Angiotensin I-converting enzyme gene insertion/deletion polymorphism and endometrial human cancer in normotensive and hypertensive women. Cancer Genet. Cytogenet..

[68] Xiao Y., Dong Z., Zhu J., You J., Fan J. (2019). Association between ACE A240T polymorphism and cancer risk: A meta-analysis. J. Int. Med. Res..

[69] Carl-McGrath S., Ebert M.P., Lendeckel U., Röcken C. (2007). Expression of the local angiotensin II system in gastric cancer may facilitate lymphatic invasion and nodal spread. Cancer Biol. Ther..

[70] Zhang K., Cheng D., Yi L., Shi H., Zhen G. (2014). Association between angiotensin I-converting enzyme gene polymorphism and susceptibility to cancer: A meta analysis. Int. J. Clin. Exp. Pathol..

[71] Loh M., Koh K.X., Yeo B.H., Song C.M., Chia K.S., Zhu F., Yeoh K.G., Hill J., Iacopetta B., Soong R. (2009). Meta-analysis of genetic polymorphisms and gastric cancer risk: Variability in associations according to race. Eur. J. Cancer.

[72] Gard P.R. (2010). Implications of the angiotensin converting enzyme gene insertion/deletion polymorphism in health and disease: A snapshot review. Int. J. Mol. Epidemiol. Genet..

[73] Ebert M.P., Lendeckel U., Westphal S., Dierkes J., Glas J., Folwaczny C., Roessner A., Stolte M., Malfertheiner P., Röcken C. (2005). The angiotensin I-converting enzyme gene insertion/deletion polymorphism is linked to early gastric cancer. Cancer Epidemiol. Biomark. Prev..

[74] Pabalan N., Jarjanazi H., Ozcelik H. (2015). Associations of the Insertion/Deletion Polymorphism in the ACE Gene and Risk of Gastric Cancer: A Meta-Analysis. J. Gastrointest. Cancer.

[75] Röcken C., Lendeckel U., Dierkes J., Westphal S., Carl-McGrath S., Peters B., Krüger S., Malfertheiner P., Roessner A., Ebert M.P. (2005). The number of lymph node metastases in gastric cancer correlates with the angiotensin I-converting enzyme gene insertion/deletion polymorphism. Clin. Cancer Res..

[76] Yang H., Cai C., Ye L., Rao Y., Wang Q., Hu D., Huang X. (2015). The relationship between angiotensin-converting enzyme gene insertion/deletion polymorphism and digestive cancer risk: Insights from a meta-analysis. J. Renin Angiotensin Aldosterone Syst..

[77] Wang Z.Y., Li H.Y., Jiang Z.P., Zhou T.B. (2018). Relationship between angiotensin-converting enzyme insertion/deletion gene polymorphism and prostate cancer susceptibility. J. Cancer Res. Ther..

[78] Said R., Jenni R., Boussetta S., Ammous F., Zouari S., Zaghbib S., Chakroun M., Derouiche A., Chebil M., Ouerhani S. (2022). Association of a common genetic variant (insertion/deletion) in ACE gene with prostate cancer susceptibility in a Tunisian population. J. Clin. Lab. Anal..

[79] Wang X., Wang S., Lin Y.W., Wu J., Chen H., Mao Y.Q., Zheng X.Y., Zhou C., Xie L.P. (2012). Angiotensin-converting enzyme insertion/deletion polymorphism and the risk of prostate cancer in the Han population of China. Med. Oncol..

[80] Medeiros R., Vasconcelos A., Costa S., Pinto D., Lobo F., Morais A., Oliveira J., Lopes C. (2004). Linkage of angiotensin I-converting enzyme gene insertion/deletion polymorphism to the progression of human prostate cancer. J. Pathol..

[81] Du J., Lan J., Yang H., Ying Q., Huang G., Mou J., Long J., Qiao Z., Hu Q. (2022). Association of angiotensin-converting enzyme insertion/deletion (ACE I/D) gene polymorphism with susceptibility to prostate cancer: An updated meta-analysis. World J. Surg. Oncol..

[82] Xie Y., You C., Chen J. (2014). An updated meta-analysis on association between angiotensin I-converting enzyme gene insertion/deletion polymorphism and cancer risk. Tumour Biol..

[83] Yigit B., Bozkurt N., Narter F., Yilmaz H., Yucebas E., Isbir T. (2007). Effects of ACE I/D polymorphism on prostate cancer risk, tumor grade and metastatis. Anticancer Res..

[84] Chung F.M., Yang Y.H., Chen C.H., Lin C.C., Shieh T.Y. (2005). Angiotensin-converting enzyme gene insertion/deletion polymorphism is associated with risk of oral precancerous lesion in betel quid chewers. Br. J. Cancer.

[85] Liu Y.T., Lin L.W., Chen C.Y., Wang C.P., Liu H.P., Houng J.Y., Chung F.M., Shieh T.Y. (2012). Polymorphism of angiotensin I-converting enzyme gene is related to oral cancer and lymph node metastasis in male betel quid chewers. Oral Oncol..

[86] Vairaktaris E., Yapijakis C., Tsigris C., Vassiliou S., Derka S., Nkenke E., Spyridonidou S., Vylliotis A., Vorris E., Ragos V. (2007). Association of angiotensin-converting enzyme gene insertion/deletion polymorphism with increased risk for oral cancer. Acta Oncol..

[87] Vylliotis A., Yapijakis C., Nkenke E., Nisyrios T., Avgoustidis D., Adamopoulou M., Ragos V., Vassiliou S., Koronellos N., Vairaktaris E. (2013). Effect of thrombosis-related gene polymorphisms upon oral cancer: A regression analysis. Anticancer Res..

[88] Vairaktaris E., Serefoglou Z., Avgoustidis D., Yapijakis C., Critselis E., Vylliotis A., Spyridonidou S., Derka S., Vassiliou S., Nkenke E. (2009). Gene polymorphisms related to angiogenesis, inflammation and thrombosis that influence risk for oral cancer. Oral Oncol..

[89] Hsieh Y.Y., Lee C.C., Chang C.C., Wang Y.K., Yeh L.S., Lin C.S. (2007). Angiotensin I-converting enzyme insertion-related genotypes and allele are associated with higher susceptibility of endometriosis and leiomyoma. Mol. Reprod. Dev..

[90] Röcken C., Neumann K., Carl-McGrath S., Lage H., Ebert M.P., Dierkes J., Jacobi C.A., Kalmuk S., Neuhaus P., Neumann U. (2007). The gene polymorphism of the angiotensin I-converting enzyme correlates with tumor size and patient survival in colorectal cancer patients. Neoplasia.

[91] Marques D., Ferreira-Costa L.R., Ferreira-Costa L.L., Correa R.D.S., Borges A.M.P., Ito F.R., Ramos C.C.O., Bortolin R.H., Luchessi A.D., Ribeiro-Dos-Santos Â. (2017). Association of insertion-deletions polymorphisms with colorectal cancer risk and clinical features. World J. Gastroenterol..

[92] Liu S.Y., Sima X., Wang C.H., Gao M. (2011). The association between ACE polymorphism and risk of colorectal cancer in a Chinese population. Clin. Biochem..

[93] Zheng X., Liu G., Cui G., Cheng M., Zhang N., Hu S. (2017). Angiotensin-Converting Enzyme Gene Deletion Polymorphism is Associated with Lymph Node Metastasis in Colorectal Cancer Patients in a Chinese Population. Med. Sci. Monit..

[94] Phukan R.K., Borah P.K., Saikia B.J., Das M., Sekhon G.S., Mahanta J. (2014). Interaction of tobacco smoking and chewing with Angiotensin converting enzyme (insertion/deletion) gene polymorphisms and risk of lung cancer in a high risk area from northeast India. Asian Pac. J. Cancer Prev..

[95] Nacak M., Nacak I., Sanli M., Ozkur M., Pektaş M., Aynacioğlu A.S. (2010). Association of angiotensin converting enzyme gene insertion/deletion polymorphism with lung cancer in Turkey. Cancer Genet. Cytogenet..

[96] Banerjee J., Gupta A., Agnihotri V., Pradhan R., Kandel R., Upadhyay A.D., Dwivedi S., Kumar L., Dey S., Dey A.B. (2021). Lung cancer in the older population: Interactive effects of angiotensin converting enzyme gene polymorphism (rs 4340 ID) and tobacco addiction in risk assessment. Indian J. Cancer.

[97] Dević Pavlić S., Ristić S., Flego V., Kapović M., Radojčić Badovinac A. (2012). Angiotensin-converting enzyme insertion/deletion gene polymorphism in lung cancer patients. Genet. Test. Mol. Biomark..

[98] Arima H., Kiyohara Y., Tanizaki Y., Nakabeppu Y., Kubo M., Kato I., Sueishi K., Tsuneyoshi M., Fujishima M., Iida M. (2006). Angiotensin I-converting enzyme gene polymorphism modifies the smoking-cancer association: The Hisayama Study. Eur. J. Cancer Prev..

[99] Peddireddy V., Badabagni S.P., Gundimeda S.D., Mundluru H.P. (2018). Association of eNOS and ACE gene polymorphisms and plasma nitric oxide with risk of non-small cell lung cancer in South India. Clin. Respir. J..

[100] Harman E., Karadeniz M., Biray C., Zengi A., Cetinkalp S., Ozgen A.G., Saygili F., Berdeli A., Gündüz C., Yilmaz C. (2009). The relation of adiponectin and tumor necrosis factor alpha levels between endothelial nitric oxide synthase, angiotensin-converting enzyme, transforming growth factor beta, and tumor necrosis factor alpha gene polymorphism in adrenal incidentalomas. J. Endocrinol. Investig..

[101] Lukic S., Nikolic A., Alempijevic T., Popovic D., Sokic Milutinovic A., Ugljesic M., Knezevic S., Milicic B., Dinic D., Radojkovic D. (2011). Angiotensin-converting enzyme gene insertion/deletion polymorphism in patients with chronic pancreatitis and pancreatic cancer. Dig. Surg..

[102] Moghimi M., Kargar S., Jafari M.A., Ahrar H., Jarahzadeh M.H., Neamatzadeh H., Sadeghizadeh- Yazdi J. (2018). Angiotensin Converting Enzyme Insertion/Deletion Polymorphism is Associated with Breast Cancer Risk: A Meta-Analysis. Asian Pac. J. Cancer Prev..

[103] Dastgheib S.A., Asadian F., Farbod M., Karimi-Zarchi M., Meibodi B., Akbarian E., Neamatzadeh H. (2021). Association of ACE I/D, -240A > T and AT1R A1166C polymorphisms with susceptibility to breast cancer: A systematic review and meta-analysis based on 35 case-control studies. Nucleosides Nucleotides Nucleic Acids.

[104] Mendizábal-Ruiz A.P., Morales J., Castro Martinez X., Gutierrez Rubio S.A., Valdez L., Vásquez-Camacho J.G., Sanchez Corona J., Moran Moguel M.C. (2011). RAS polymorphisms in cancerous and benign breast tissue. J. Renin Angiotensin Aldosterone Syst..

[105] Singh A., Srivastava N., Amit S., Prasad S.N., Misra M.P., Ateeq B. (2018). Association of AGTR1 (A1166C) and ACE (I/D) Polymorphisms with Breast Cancer Risk in North Indian Population. Transl. Oncol..

[106] Namazi S., Monabati A., Ardeshir-Rouhani-Fard S., Azarpira N. (2010). Association of angiotensin I converting enzyme (insertion/deletion) and angiotensin II type 1 receptor (A1166C) polymorphisms with breast cancer prognostic factors in Iranian population. Mol. Carcinog..

[107] Namazi S., Daneshian A., Mohammadianpanah M., Jafari P., Ardeshir-Rouhani-Fard S., Nasirabadi S. (2013). The impact of renin-angiotensin system, angiotensin I converting enzyme (insertion/deletion), and angiotensin II type 1 receptor (A1166C) polymorphisms on breast cancer survival in Iran. Gene.

[108] Yaren A., Turgut S., Kursunluoglu R., Oztop I., Turgut G., Degirmencioglu S., Kelten C., Erdem E. (2007). Insertion/deletion polymorphism of the angiotensin I-converting enzyme gene in patients with breast cancer and effects on prognostic factors. J. Investig. Med..

[109] Yaren A., Turgut S., Kursunluoglu R., Oztop I., Turgut G., Kelten C., Erdem E. (2006). Association between the polymorphism of the angiotensin-converting enzyme gene and tumor size of breast cancer in premenopausal patients. Tohoku J. Exp. Med..

[110] Van der Knaap R., Siemes C., Coebergh J.W., van Duijn C.M., Hofman A., Stricker B.H. (2008). Renin-angiotensin system inhibitors, angiotensin I-converting enzyme gene insertion/deletion polymorphism, and cancer: The Rotterdam Study. Cancer.

[111] González-Zuloeta Ladd A.M., Arias Vásquez A., Sayed-Tabatabaei F.A., Coebergh J.W., Hofman A., Njajou O., Stricker B., van Duijn C. (2005). Angiotensin-converting enzyme gene insertion/deletion polymorphism and breast cancer risk. Cancer Epidemiol. Biomark. Prev..

[112] Yuan F., Zhang L.S., Li H.Y., Liao M., Lv M., Zhang C. (2013). Influence of angiotensin I-converting enzyme gene polymorphism on hepatocellular carcinoma risk in China. DNA Cell Biol..

[113] Zha Y., Gan P., Liu Q., Tan J. (2015). Relationship between polymorphism of angiotensin-converting enzyme gene insertion/deletion and risk of hepatocellular carcinoma in a Chinese Dai population. J. Renin Angiotensin Aldosterone Syst..

[114] Alves Corrêa S.A., Ribeiro de Noronha S.M., Nogueira-de-Souza N.C., Valleta de Carvalho C., Massad Costa A.M., Juvenal Linhares J., Vieira Gomes M.T., Guerreiro da Silva I.D. (2009). Association between the angiotensin-converting enzyme (insertion/deletion) and angiotensin II type 1 receptor (A1166C) polymorphisms and breast cancer among Brazilian women. J. Renin Angiotensin Aldosterone Syst..

[115] Keshavarzi F., Teimoori B., Farzaneh F., Mokhtari M., Najafi D., Salimi S. (2019). Association of ACE I/D and AGTR1 A1166C Gene Polymorphisms and Risk of Uterine Leiomyoma: A Case-Control Study. Asian Pac. J. Cancer Prev..

[116] Srivastava K., Srivastava A., Mittal B. (2010). Angiotensin I-converting enzyme insertion/deletion polymorphism and increased risk of gall bladder cancer in women. DNA Cell Biol..

[117] Benenemissi I.H., Sifi K., Sahli L.K., Semmam O., Abadi N., Satta D. (2019). Angiotensin-converting enzyme insertion/deletion gene polymorphisms and the risk of glioma in an Algerian population. Pan Afr. Med. J..

[118] Pandith A.A., Qasim I., Zahoor W., Shah P., Bhat A.R. (2018). ACE I/D sequence variants but not MTHFR C677T, is strongly linked to malignant glioma risk and its variant DD genotype may act as a promising predictive biomarker for overall survival of glioma patients. Gene.

[119] Lian M., Jiang H., Wang H., Guo S. (2015). Angiotensin-converting enzyme insertion/deletion gene polymorphisms is associated with risk of glioma in a Chinese population. J. Renin Angiotensin Aldosterone Syst..

[120] De Martino M., Klatte T., Schatzl G., Waldert M., Remzi M., Haitel A., Kramer G., Marberger M. (2011). Insertion/deletion polymorphism of angiotensin I-converting enzyme gene is linked with chromophobe renal cell carcinoma. Urology.

[121] Ali S.H.B., Bangash K.S., Rauf A., Younis M., Anwar K., Khurram R., Khawaja M.A., Azam M., Qureshi A.A., Akhter S. (2017). Identification of novel potential genetic predictors of urothelial bladder carcinoma susceptibility in Pakistani population. Fam. Cancer.

[122] Yapijakis C., Gintoni I., Charalampidou S., Angelopoulou A., Papakosta V., Vassiliou S., Chrousos G.P. (2023). Angiotensinogen, Angiotensin-Converting Enzyme, and Chymase Gene Polymorphisms as Biomarkers for Basal Cell Carcinoma Susceptibility. Adv. Exp. Med. Biol..

[123] Yapijakis C., Koronellos N., Spyridonidou S., Vylliotis A., Avgoustidis D., Goutas N., Vlachodimitropoulos D., Vairaktaris E. (2013). Association of angiotensin-converting enzyme gene insertion/deletion polymorphism with decreased risk for basal cell carcinoma. Arch. Dermatol. Res..

[124] Koronellos N., Yapijakis C., Katoulis A., Avgoustidis D., Vylliotis A., Papakosta V., Diamantopoulou S., Zografos O., Vairaktari G., Vairaktaris E. (2021). Association study indicates combined effect of interleukin-10 and angiotensin-converting enzyme in basal cell carcinoma development. Arch. Dermatol. Res..

[125] Hajek D., Tomiska M., Krahulcova E., Druckmuller M., Florianova M., Izakovicova-Holla L., Vacha J. (2003). I/D ACE gene polymorphism in survival of leukemia patients—Hypothesis and pilot study. Med. Hypotheses.

[126] Kumbul Y., Hekimler Öztürk K., Yasan H., Akın V., Sivrice M.E., Caner F. (2023). Angiotensin-converting enzyme insertion/deletion gene polymorphism in patients with laryngeal cancer. Acta Otorhinolaryngol. Ital..

[127] Altas M., Bayrak O.F., Serefhan A., Silav G., Coskun K.K., Cerci A., Isik N., Elmaci I. (2013). Investigation of ACE genome insertion/deletion correlation with immunohistochemical profile in pituitary adenomas. Turk. Neurosurg..

[128] Correa-Noronha S.A., Noronha S.M., Alecrim C., Mesquita Ade C., Brito G.S., Junqueira M.G., Leite D.B., Carvalho C.V., Silva I.D. (2012). Association of angiotensin-converting enzyme I gene I/D polymorphism with endometrial but not with ovarian cancer. Gynecol. Endocrinol..

[129] Vigano A., Trutschnigg B., Kilgour R.D., Hamel N., Hornby L., Lucar E., Foulkes W., Tremblay M.L., Morais J.A. (2009). Relationship between angiotensin-converting enzyme gene polymorphism and body composition, functional performance, and blood biomarkers in advanced cancer patients. Clin. Cancer Res..

[130] Ding P., Yang Y., Ding S., Sun B. (2015). Synergistic association of six well-characterized polymorphisms in three genes of the renin-angiotensin system with breast cancer among Han Chinese women. J. Renin Angiotensin Aldosterone Syst..

[131] Fishchuk L.E., Gorovenko N.G. (2013). Genetic polymorphisms of the renin-angiotensin system in breast cancer patients. Exp. Oncol..

[132] Röcken C., Röhl F.W., Diebler E., Lendeckel U., Pross M., Carl-McGrath S., Ebert M.P. (2007). The angiotensin II/angiotensin II receptor system correlates with nodal spread in intestinal type gastric cancer. Cancer Epidemiol. Biomark. Prev..

[133] Gan L., Liu X., Wu Z., Huang M., Zhang X., Guo W. (2015). Angiotensin-converting enzyme insertion/deletion polymorphism and gastric cancer: A systematic review and meta-analysis. Int. J. Clin. Exp. Med..

[134] Goto Y., Ando T., Nishio K., Ishida Y., Kawai S., Goto H., Hamajima N. (2005). The ACE gene polymorphism is associated with the incidence of gastric cancer among H. pylori seropositive subjects with atrophic gastritis. Asian Pac. J. Cancer Prev..

[135] Papaggelopoulos J., Angelopoulou A., Avgoustidis D., Koronellos N., Derka S., Vassiliou S., Yapijakis C. (2019). Association of Polymorphisms in the Genes of Angiotensinogen and Angiotensin Receptors with Risk for Basal Cell Carcinoma. Anticancer Res..

[136] Samara M., Papathanassiou M., Farmakioti I., Anagnostou M., Satra M., Mitrakas L., Anastasiou D., Chasiotis G., Christopoulos A., Anagnostou A. (2021). Renin-Angiotensin System Single Nucleotide Polymorphisms Are Associated with Bladder Cancer Risk. Curr. Oncol..

[137] Huhn S., Bevier M., Rudolph A., Pardini B., Naccarati A., Hein R., Hoffmeister M., Vodickova L., Novotny J., Brenner H. (2012). Shared ancestral susceptibility to colorectal cancer and other nutrition related diseases. BMC Med. Genet..

[138] González-Zuloeta Ladd A.M., Arias Vásquez A., Siemes C., Yazdanpanah M., Coebergh J.W., Hofman A., Stricker B.H., van Duijn C.M. (2007). Differential roles of Angiotensinogen and Angiotensin Receptor type 1 polymorphisms in breast cancer risk. Breast Cancer Res. Treat..

[139] Andreotti G., Boffetta P., Rosenberg P.S., Berndt S.I., Karami S., Menashe I., Yeager M., Chanock S.J., Zaridze D., Matteev V. (2010). Variants in blood pressure genes and the risk of renal cell carcinoma. Carcinogenesis.

[140] Vasků A., Vokurka J., Bienertová-Vasků J. (2009). Obesity-related genes variability in Czech patients with sporadic colorectal cancer: Preliminary results. Int. J. Color. Dis..

[141] League S., Hooper W.C. (2005). Molecular diagnostics of inherited thrombosis. Clin. Lab. Sci..

[142] Lim M.Y., Deal A.M., Kim S., Musty M.D., Conard J., Simioni P., Dutrillaux F., Eid S.S., Middeldorp S., Halbmayer W.M. (2016). Thrombophilic risk of individuals with rare compound factor V Leiden and prothrombin G20210A polymorphisms: An international case series of 100 individuals. Eur. J. Haematol..

[143] Poort S.R., Rosendaal F.R., Reitsma P.H., Bertina R.M. (1996). A common genetic variation in the 3′-untranslated region of the prothrombin gene is associated with elevated plasma prothrombin levels and an increase in venous thrombosis. Blood.

[144] Tormene D., Beltramello P., Perlati M., Brandolin B., Barbar S., De Toffoli G., Simioni P. (2009). The risk of cancer progression in women with gynecological malignancies and thrombophilic polymorphisms: A pilot case-control study. Clin. Appl. Thromb. Hemost..

[145] Hanafy A.S., Alaa F.A., Randa M.H. (2016). Association of thrombogenic genes polymorphisms with hepatocellular carcinoma in HCV Egyptian patients. Gene.

[146] Baghad I., Erguibi D., Chehab F., Nadifi S. (2017). Risk of colorectal cancer and clotting factor gene polymorphisms in Moroccan Population. Int. J. Adv. Res..

[147] Vossen C.Y., Hoffmeister M., Chang-Claude J.C., Rosendaal F.R., Brenner H. (2011). Clotting factor gene polymorphisms and colorectal cancer risk. J. Clin. Oncol..

[148] Leite A.R., Borges-Canha M., Cardoso R., Neves J.S., Castro-Ferreira R., Leite-Moreira A. (2020). Novel Biomarkers for Evaluation of Endothelial Dysfunction. Angiology.

[149] Deanfield J.E., Halcox J.P., Rabelink T.J. (2007). Endothelial function and dysfunction: Testing and clinical relevance. Circulation.

[150] Chia P.Y., Teo A., Yeo T.W. (2020). Overview of the Assessment of Endothelial Function in Humans. Front. Med..

[151] Powrózek T., Mlak R., Brzozowska A., Mazurek M., Gołębiowski P., Małecka-Massalska T. (2019). Relationship Between -2028 C/T SELP Gene Polymorphism, Concentration of Plasma P-Selectin and Risk of Malnutrition in Head and Neck Cancer Patients. Pathol. Oncol. Res..

[152] Tan B.H., Fladvad T., Braun T.P., Vigano A., Strasser F., Deans D.A., Skipworth R.J., Solheim T.S., Damaraju S., Ross J.A. (2012). P-selectin genotype is associated with the development of cancer cachexia. EMBO Mol. Med..

[153] Avan A., Avan A., Le Large T.Y., Mambrini A., Funel N., Maftouh M., Ghayour-Mobarhan M., Cantore M., Boggi U., Peters G.J. (2014). AKT1 and SELP polymorphisms predict the risk of developing cachexia in pancreatic cancer patients. PLoS ONE.

[154] Zakariya B.F., Almohaidi A.M.S., Şimşek S.A., Al-Waysi S.A., Al-Dabbagh W.H., Kamal A.M. (2022). The relationship of E-selectin singlenucleotide polymorphisms with breast cancer in Iraqi Arab women. Genom. Inform..

[155] Turpin A., Labreuche J., Fléjou J.F., Andre T., de Gramont A., Hebbar M. (2019). Prognostic factors in patients with stage II colon cancer: Role of E-selectin gene polymorphisms. Dig. Liver Dis..

[156] Alessandro R., Seidita G., Flugy A.M., Damiani F., Russo A., Corrado C., Colomba P., Gullotti L., Buettner R., Bruno L. (2007). Role of S128R polymorphism of E-selectin in colon metastasis formation. Int. J. Cancer.

[157] Naidu R., Har Y.C., Taib N.A. (2011). Polymorphic variant Ser128Arg of E-Selectin is associated with breast cancer risk and high grade tumors. Onkologie.

[158] Golnarnik G., Mashayekhi F., Saedi H.S. (2016). The E-selectin S149R polymorphisms in breast cancer in a northern Iran population. Cell. Mol. Biol..

[159] Xia H.Z., Du W.D., Wu Q., Chen G., Zhou Y., Tang X.F., Tang H.Y., Liu Y., Yang F., Ruan J. (2012). E-selectin rs5361 and FCGR2A rs1801274 variants were associated with increased risk of gastric cancer in a Chinese population. Mol. Carcinog..

[160] Liarmakopoulos E., Gazouli M., Aravantinos G., Theodoropoulos G., Rizos S., Vaiopoulou A., Kouraklis G., Nikiteas N. (2013). E-Selectin S128R gene polymorphism in gastric cancer. Int. J. Biol. Markers.

[161] Panoussopoulos G.S., Theodoropoulos G., Michalopoulos N.V., Gazouli M., Flessas J., Taka S., Stamopoulos P., Manouras A., Zografos G.C. (2010). Analysis of E-Selectin S128R gene polymorphism in pancreatic cancer. J. Surg. Oncol..

[162] Lu Z.H., Gu X.J., Shi K.Z., Li X., Chen D.D., Chen L. (2016). Association between genetic polymorphisms of inflammatory response genes and the risk of ovarian cancer. J. Formos. Med. Assoc..

[163] Kontogianni P., Zambirinis C.P., Theodoropoulos G., Gazouli M., Michalopoulos N.V., Flessas J., Liberi M., Zografos G.C. (2013). The impact of the stromal cell-derived factor-1-3′A and E-selectin S128R polymorphisms on breast cancer. Mol. Biol. Rep..

[164] Cheng D.Y., Hao Y.W., Zhou W.L., Ma Y.R. (2014). E-selectin S128R polymorphism is associated with cancer risk: A meta-analysis. Asian Pac. J. Cancer Prev..

[165] Calabrò P., Gragnano F., Golia E., Grove E.L. (2018). von Willebrand Factor and Venous Thromboembolism: Pathogenic Link and Therapeutic Implications. Semin. Thromb. Hemost..

[166] Qian D., Liu H., Wang X., Ge J., Luo S., Patz E.F., Moorman P.G., Su L., Shen S., Christiani D.C. (2019). Potentially functional genetic variants in the complement-related immunity gene-set are associated with non-small cell lung cancer survival. Int. J. Cancer.

[167] Arandi N., Talei A., Erfani N., Ghaderi A. (2008). Intercellular adhesion molecule-1 genetic markers (+241G/A and +469A/G) in Iranian women with breast cancer. Cancer Genet. Cytogenet..

[168] Yilmaz U., Zeybek U., Kahraman O.T., Kafadar A.M., Toptas B., Yamak N., Celik F., Yaylim I. (2013). Investigation of ICAM-1 and β3 integrin gene variations in patients with brain tumors. Asian Pac. J. Cancer Prev..

[169] Feng Y., Li X., Ma Q., Zhang S., Zhu M., Li S., Fang L., Tian J., Sun L. (2021). Intercellular Adhesion Molecule-1 Gene Polymorphisms and Susceptibility to Cervical Cancer in the Northern Chinese Han Population. Front. Genet..

[170] Liu A., Wan A., Feng A., Rui R., Zhou B. (2018). ICAM-1 gene rs5498 polymorphism decreases the risk of coronary artery disease. Medicine.

[171] Qiu Z., Wang Y., Zhang Z., Qin R., Peng Y., Tang W., Xi Y., Tian G., Zhang Y. (2022). Roles of intercellular cell adhesion molecule-1 (ICAM-1) in colorectal cancer: Expression, functions, prognosis, tumorigenesis, polymorphisms and therapeutic implications. Front. Oncol..

[172] Cheng D., Liang B. (2015). Intercellular Adhesion Molecule-1 (ICAM-1) Polymorphisms and Cancer Risk: A Meta-Analysis. Iran. J. Public Health.

[173] Tang W., Wang Y., Chen Y., Gu H., Chen S., Kang M. (2015). Polymorphisms in the intercellular adhesion molecule 1 gene and cancer risk: A meta-analysis. Int. J. Clin. Exp. Med..

[174] Lin C.W., Chuang C.Y., Tang C.H., Chang J.L., Lee L.M., Lee W.J., Chow J.M., Yang S.F., Chien M.H. (2013). Combined effects of icam-1 single-nucleotide polymorphisms and environmental carcinogens on oral cancer susceptibility and clinicopathologic development. PLoS ONE.

[175] Liu L.B., Liu T., Xin F.Z. (2017). Correlations of ICAM-1 gene polymorphisms with susceptibility and multidrug resistance in colorectal cancer in a Chinese population. Medicine.

[176] Qiu Z., Xie Z., Qin R., Chen M., He H., Zhang Z., Wang Y., Hong M., Tang W., Xi Y. (2021). Evaluation of ICAM-1 rs5498 and rs3093030 Polymorphisms in Chinese Patients with Colorectal Cancer. DNA Cell Biol..

[177] Theodoropoulos G., Papaconstantinou I., Felekouras E., Nikiteas N., Karakitsos P., Panoussopoulos D., Lazaris A., Patsouris E., Bramis J., Gazouli M. (2006). Relation between common polymorphisms in genes related to inflammatory response and colorectal cancer. World J. Gastroenterol..

[178] Novikov V.V., Shumilova S.V., Novikov D.V., Kalugin A.V., Fomina S.G., Karaulov A.V. (2016). Genetic Instability in Locus rs5498 E469K (A/G) of ICAM-1 Gene in Patients with Colorectal Cancer and Breast Cancer. Bull. Exp. Biol. Med..

[179] Wang Q.L., Li B.H., Liu B., Liu Y.B., Liu Y.P., Miao S.B., Han Y., Wen J.K., Han M. (2009). Polymorphisms of the ICAM-1 exon 6 (E469K) are associated with differentiation of colorectal cancer. J. Exp. Clin. Cancer Res..

[180] Tian M.M., Sun Y., Li Z.W., Wu Y., Zhao A.L., Li J.Y. (2012). Polymorphisms of ICAM-1 are associated with gastric cancer risk and prognosis. World J. Gastroenterol..

[181] Thanopoulou E., Kotzamanis G., Pateras I.S., Ziras N., Papalambros A., Mariolis-Sapsakos T., Sigala F., Johnson E., Kotsinas A., Scorilas A. (2012). The single nucleotide polymorphism g.1548A > G (K469E) of the ICAM-1 gene is associated with worse prognosis in non-small cell lung cancer. Tumor Biol..

[182] Cai G., Ma X., Zou W., Huang Y., Zhang J., Wang D., Chen B. (2014). Prediction value of intercellular adhesion molecule-1 gene polymorphisms for epithelial ovarian cancer risk, clinical features, and prognosis. Gene.

[183] Wang S.S., Hsieh M.J., Ou Y.C., Chen C.S., Li J.R., Hsiao P.C., Yang S.F. (2014). Impacts of ICAM-1 gene polymorphisms on urothelial cell carcinoma susceptibility and clinicopathologic characteristics in Taiwan. Tumor Biol..

[184] Chen T.P., Lee H.L., Huang Y.H., Hsieh M.J., Chiang W.L., Kuo W.H., Chou M.C., Yang S.F., Yeh C.B. (2016). Association of intercellular adhesion molecule-1 single nucleotide polymorphisms with hepatocellular carcinoma susceptibility and clinicopathologic development. Tumor Biol..

[185] Chen H., Hernandez W., Shriver M.D., Ahaghotu C.A., Kittles R.A. (2006). ICAM gene cluster SNPs and prostate cancer risk in African Americans. Hum. Genet..

[186] Sun Y.H., Yang S.F., Liu Y.F., Ko J.L., Wu C.H., Wu T.F., Wang P.H. (2016). Single-Nucleotide Polymorphisms and Haplotypes of Intercellular Adhesion Molecule-1 in Uterine Cervical Carcinogenesis in Taiwanese Women. Reprod. Sci..

[187] Burim R.V., Teixeira S.A., Colli B.O., Peria F.M., Tirapelli L.F., Marie S.K., Malheiros S.M., Oba-Shinjo S.M., Gabbai A.A., Lotufo P.A. (2009). ICAM-1 (Lys469Glu) and PECAM-1 (Leu125Val) polymorphisms in diffuse astrocytomas. Clin. Exp. Med..

[188] Zhang X., Huang J., Bai J., Lu W., Zhang M., Mei H. (2016). Association of Polymorphisms in Intercellular Adhesion Molecule 1 (ICAM-1) Gene with Cancer Susceptibility: A Meta-Analysis of 14 Case-Control Studies. Med. Sci. Monit..

[189] He L., Wang S., Ma X. (2021). The Influence of ICAM1 3′UTR Gene Polymorphism on the Occurrence and Metastasis of Primary Liver Cancer. Biomed. Res. Int..

[190] Ghazy A.A., Elsheredy H.G., Abouelella A.M., Rashwan E.K., Khaled B.E.A., Elsheredy A.G. (2020). Significance of Intracellular Adhesion Molecule-1 Polymorphism and IP-10 among Breast Cancer Patients. Egypt. J. Immunol..

[191] Ghazy A.A., El-Etreby N.M. (2016). Relevance of HLA-DP/DQ and ICAM-1 SNPs among Ovarian Cancer Patients. Front. Immunol..

[192] Dadgar Pakdel F., Keramatipour M., Noorbakhsh F., Talebi S., Vodjgani M. (2015). Investigating the Effect of rs3783605 Single-nucleotide Polymorphism on the Activity of VCAM-1 Promoter in Human Umbilical Vein Endohelial Cells. Iran. J. Allergy Asthma Immunol..

[193] Miaskowski C., Dodd M., Paul S.M., West C., Hamolsky D., Abrams G., Cooper B.A., Elboim C., Neuhaus J., Schmidt B.L. (2013). Lymphatic and angiogenic candidate genes predict the development of secondary lymphedema following breast cancer surgery. PLoS ONE.

[194] Wang S.S., Carreon J.D., Hanchard B., Chanock S., Hisada M. (2009). Common genetic variants and risk for non-Hodgkin lymphoma and adult T-cell lymphoma/leukemia in Jamaica. Int. J. Cancer.

[195] Yu G.I., Jun S.E., Shin D.H. (2017). Associations of VCAM-1 gene polymorphisms with obesity and inflammation markers. Inflamm. Res..

[196] Andrew A.S., Gui J., Hu T., Wyszynski A., Marsit C.J., Kelsey K.T., Schned A.R., Tanyos S.A., Pendleton E.M., Ekstrom R.M. (2015). Genetic polymorphisms modify bladder cancer recurrence and survival in a USA population-based prognostic study. BJU Int..

[197] Ehrenfeld P., Cordova F., Duran W.N., Sanchez F.A. (2019). S-nitrosylation and its role in breast cancer angiogenesis and metastasis. Nitric Oxide.

[198] Nastri C.O., Martins Wde P., Reis F.J., Ferriani R.A. (2008). Breast cancer and endothelial dysfunction. Rev. Assoc. Med. Bras..

[199] Choudhari S.K., Chaudhary M., Bagde S., Gadbail A.R., Joshi V. (2013). Nitric oxide and cancer: A review. World J. Surg. Oncol..

[200] Knowles J., Loizidou M., Taylor I. (2005). Endothelin-1 and angiogenesis in cancer. Curr. Vasc. Pharmacol..

[201] Cazaubon S., Deshayes F., Couraud P.O., Nahmias C. (2006). Endothelin-1, angiotensin II and cancer. Med. Sci..

[202] Zhao Y., Chen X., Cai L., Yang Y., Sui G., Fu S. (2010). Angiotensin II/angiotensin II type I receptor (AT1R) signaling promotes MCF-7 breast cancer cells survival via PI3-kinase/Akt pathway. J. Cell. Physiol..

[203] Deshayes F., Nahmias C. (2005). Angiotensin receptors: A new role in cancer?. Trends Endocrinol. Metab..

[204] Franchini M., Mannucci P.M. (2012). Thrombin and cancer: From molecular basis to therapeutic implications. Semin. Thromb. Hemost..

[205] Rickles F.R., Patierno S., Fernandez P.M. (2003). Tissue factor, thrombin, and cancer. Chest.

[206] Chang Y., Wei W. (2015). Angiotensin II in inflammation, immunity and rheumatoid arthritis. Clin. Exp. Immunol..

[207] Harjunpää H., Llort Asens M., Guenther C., Fagerholm S.C. (2019). Cell Adhesion Molecules and Their Roles and Regulation in the Immune and Tumor Microenvironment. Front. Immunol..

[208] Kobayashi H., Boelte K.C., Lin P.C. (2007). Endothelial cell adhesion molecules and cancer progression. Curr. Med. Chem..

